# Bile acid accumulation induced by miR-122 deficiency in liver parenchyma promotes cancer cell growth in hepatocellular carcinoma

**DOI:** 10.1016/j.omtn.2025.102560

**Published:** 2025-05-14

**Authors:** Jia-Hui Huang, Yi-Hang Li, Juan-Zhen Hong, Ruo-Nan Li, Ruizhi Wang, Zi-Qi Chen, Song-Yang Li, Ying-Lei Chi, Jin-Yu Huang, Ying Zhu

**Affiliations:** 1 MOE Key Laboratory of Gene Function and Regulation, Guangdong Province Key Laboratory of Pharmaceutical Functional Genes, Innovation Center for Evolutionary Synthetic Biology, School of Life Sciences, State Key Laboratory of Oncology in Southern China, Sun Yat-sen University, 135 Xin Gang Xi Road, Guangzhou 510275, P.R. China; 2 Department of Laboratory Medicine, The First Affiliated Hospital, Sun Yat-sen University, Guangzhou 510080, P.R. China

**Keywords:** MT: Non-coding RNAs, bile acid, miR-122, HSD3B7, HCC, paracancerous tissues

## Abstract

Liver is the central player in maintaining metabolic homeostasis of bile acids (BAs), but how BA is tightly controlled is still largely unknown, and the role of BAs in the development of hepatocellular carcinoma (HCC) remains controversial. Here, we discovered that elevated hepatic BAs were associated with miR-122 downregulation during liver regeneration, steatosis, and fibrosis. *In vivo* mouse models showed that miR-122 deficiency of liver parenchymal cells (hepatocytes) in paracancerous tissues resulted in significantly increased BA levels and altered hepatic BA spectrum, thus promoting liver tumor burden, which could be abated by administration of BA sequestrant. Mechanistically, miR-122 attenuated BA production by directly targeting BA synthesis gene *HSD3B7*, thereby inhibiting cancer cell proliferation and HCC growth. Overexpression of HSD3B7 in hepatocytes abolished the inhibitory effect of intrahepatic delivery of miR-122 on cancer cell proliferation in c-Myc/sgTP53-induced HCC model. Consistently, lower miR-122 was associated with elevated levels of BA and HSD3B7 protein in paracancerous tissues from HCC patients and also associated with worse overall survival of HCC patients. These findings provide novel insights into the roles of miR-122-mediated BA regulatory network of liver parenchymal cells of tumor microenvironment during HCC progression, which may provide attractive therapeutic targets for HCC.

## Graphical abstract

**Figure:**
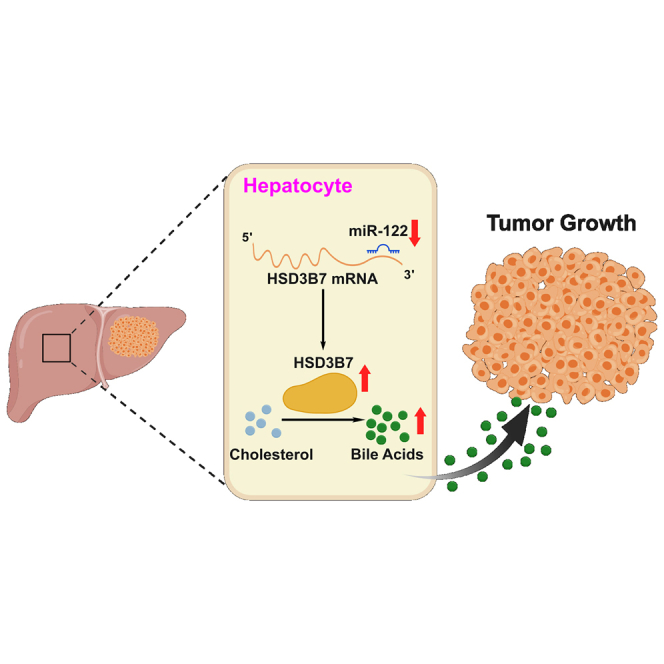

## Teaser

The control of BA homeostasis is unclear, and its role in HCC development remains controversial. Zhu and colleagues demonstrate that miR-122 deficiency in hepatocytes induces BA production by directly upregulating *HSD3B7*, thereby facilitating cancer cell proliferation and HCC growth. Their findings identify miR-122-HSD3B7-BAs axis as potential therapeutic targets for HCC.

## Introduction

Originally identified as amphipathic steroid metabolites to facilitate the absorption of lipids and lipid-soluble nutrients from intestine, bile acids (BAs) are now known as endocrine signaling molecules to regulate a variety of physiological processes including lipid and carbohydrate metabolism,^1^ energy expenditure,^2^ immunity,^3^^,^^4^ and gut microbiota homeostasis.^5^ However, how BA is tightly controlled is still largely unknown. Liver is known as the central player in maintaining metabolic homeostasis of BAs. In hepatocytes, primary BAs are directly synthesized from cholesterol by at least 17 liver enzymes via the classical or alternative pathways, then some of which undergo conjugation with glycine and taurine.^6^ BA-sensing receptor FXR, which was considered as the master regulator of BA homeostasis, controlled synthesis, uptake, and secretion of hepatic BAs by negative feedback mechanisms through inhibiting the expression of BA synthesis enzymes including CYP7A1 and CYP8B1 and BA import transporters like NTCP and ASBT, along with the induction of BA export pumps including BSEP and OSTα/β.^7^ Nevertheless, liver-specific knockout of FXR only slightly increased BA pool size rather than hepatic BAs.^8^^,^^9^ Obviously, it is highly worthwhile to identify key players that elaborately control BA homeostasis in liver.

Hepatocellular carcinoma (HCC) is one of the most lethal cancers worldwide, which typically develops in people with chronic hepatitis and cirrhosis caused by hepatitis virus infection, non-alcoholic fatty liver disease, alcohol addition, and exposure to dietary toxin such as aflatoxins and aristolochic acids.^10^ Although liver is an important organ for BA metabolism, the role of BAs in the occurrence and progression of HCC remains controversial. An association of increased amounts of hepatic BAs with a bleak prognosis was observed in HCC patients.^11^ However, a recent study in multi-omics profiles discovered that most key proteins in BA metabolism were downregulated in the tumor tissues of HBV-related HCC,^12^ indicating that cancer cells might lose their liver-specific BA metabolic function during hepatocarcinogenesis. Moreover, from a mechanistic point of view, whether BA is a promoter or inhibitor of HCC development is also inconclusive. It has been reported that the primary-to-secondary BA conversion mediated by gut microbiome controls a chemokine-dependent accumulation of hepatic natural killer T (NKT) cells and anti-tumor immunity in the liver, against both primary and metastatic liver tumors.^13^ Interestingly, increased level of secondary BA deoxycholic acid (DCA) produced by gut microbiota promotes obesity-associated HCC development by provoking the senescence-related secretory phenotype of hepatic stellate cells via COX2-PGE2 signaling axis.^14^ Elevated BA production caused by activating Hippo signaling or the loss of Sirt5 promotes hepatocarcinogenesis via controlling liver growth or creating an immunosuppressive microenvironment, respectively.^15^^,^^16^ Therefore, extensive investigations are required to clarify these contradictions and delineate the molecular basis underlying the BA metabolic heterogeneity in HCC progression.

miR-122, the most abundant miRNA in the adult liver, is implicated as a central player in liver physiology and pathology, such as lipid metabolism, infection of hepatitis B virus and hepatitis C virus, hepatic fibrosis, and HCC development,^17^^,^^18^ by targeting a number of genes, including Agpat1,^19^ CCNG1,^20^ Klf6,^21^ Ccl2,^19^ and IGF1R.^22^ However, it remains unclear whether miR-122 modulates BA metabolism and thus affects the disease development. Moreover, for two decades, most studies have been focusing on the functions of miR-122 in HCC tumor cells, rarely addressing how miR-122 in liver parenchyma (which is represented by hepatocytes) affects tumor cell behavior and tumor progression. Here, we explored a mechanism by which miR-122 in liver parenchymal cells, the largest population of non-cancer cells in tumor microenvironment of HCC, regulates BA metabolism to affect tumor growth.

In this study, we showed that in many physiological and pathological conditions of liver, reduced miR-122 was expressed concomitantly with elevated BAs. Loss of miR-122 in hepatocytes promoted BA biosynthesis by enhancing the expression of its target gene *Hsd3b7*, thereby facilitating tumor growth of HCC cells. Consistently, downregulation of miR-122 was significantly positively correlated with elevation of BAs and HSD3B7 protein in paracancerous tissues from HCC patients, and lower miR-122 level in paracancerous tissues was associated with poorer overall survival of HCC patients. Our findings suggest the role of miR-122-mediated BA regulatory network in hepatocarcinogenesis and provide the miR-122-HSD3B7-BA regulatory axis as an attractive target for HCC therapy.

## Results

### Elevated hepatic BA levels are associated with decreased levels of miR-122 in numerous physiological and pathological processes of liver

**Figure 1:**
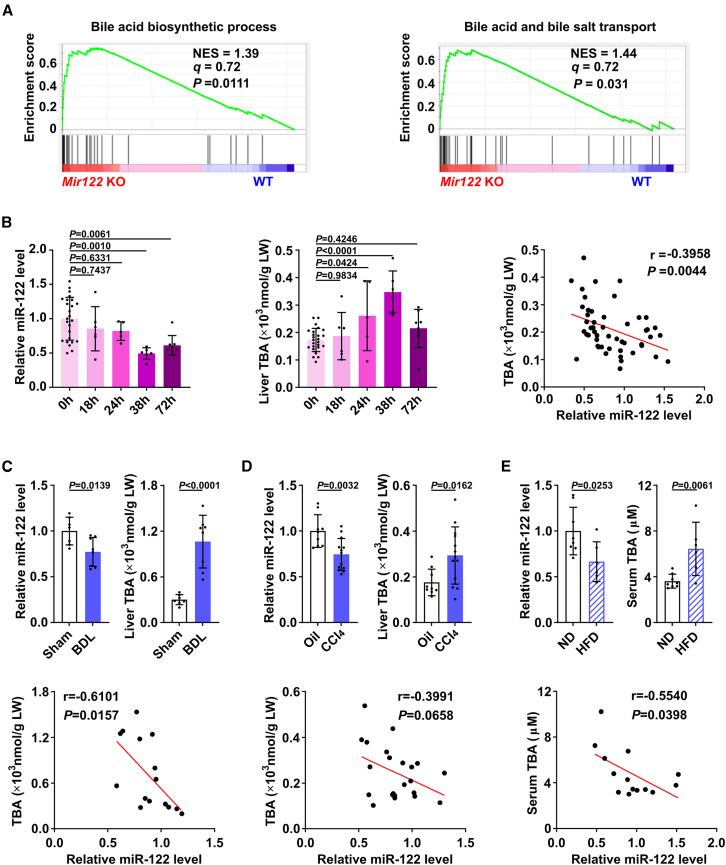
Elevated hepatic BA levels are associated with decreased levels of miR-122 in numerous physiological and pathological processes of liver (A) The BA biosynthetic process and BA and bile salt transport pathway were significantly enriched in *Mir122* KO livers of mice. The *q* and *p* values were determined by GSEA, and data were from GSE97060. (B) Significant negative correlation between hepatic miR-122 and TBA during liver regeneration (n = 5–8 mice per group). (Left) The expression pattern of hepatic miR-122 was examined by qPCR in PH model. (Middle) Hepatic TBA level was detected in PH mice. (Right) The correlation between hepatic BAs and liver miR-122 in PH model. 0 h denotes two-thirds of the liver that was surgically removed; 18, 24, 38, and 72 h denote livers that were obtained at the indicated time points after PH. (C, D) Elevated hepatic BAs were associated with the downregulation of miR-122 in fibrotic livers (n = 7–11 mice per group). Samples were collected from BDL-treated (C) or CCl_4_-treated mice (D). (Upper) miR-122 was decreased, and BAs were increased in mouse fibrotic livers. (Lower) The correlation between hepatic BAs and miR-122 in BDL model and CCl_4_-treated mice. (E) Significant correlation between downregulation of hepatic miR-122 and elevation of serum TBA in HFD-fed mice (n = 6–8 mice per group). (Upper) Hepatic miR-122 expression was decreased and serum BAs were elevated in HFD mice. (Lower) The correlation between hepatic miR-122 and serum BAs in HFD model. For (B–E), U6 was used as an internal control for miR-122, and the mean levels in the 0 h, Sham, Oil, or ND group were set as relative level 1, respectively. For (B–D), total hepatic BA levels were normalized to liver weight (LW) in each mouse. Data are presented as mean ± SD (B, left and middle panels; C–E, upper panels); *p* values were assessed by one-way ANOVA (B, left and middle panels) or unpaired Student’s t test (C–E, upper panels) or Pearson correlation coefficient (B, right panel; C–E, lower panels).

To evaluate whether miR-122 is involved in BA metabolism in the liver, gene set enrichment analysis (GSEA) on the transcriptome profiles of livers from *Mir122* KO mice and their wild-type littermates was performed. As shown, genes in BA biosynthesis process and BA and bile salt transport were significantly enriched in *Mir122* KO livers compared with wild-type livers (Figure 1A). Then we investigated the BA levels and miR-122 levels in a number of physiological and pathological processes of liver. In a physiological liver regeneration model by conducting PH (partial hepatectomy) in mice, the hepatic level of miR-122 was negatively correlated with the level of hepatic total BAs (TBAs). Specifically, the expression of miR-122 in the liver had a large decrease at 38 h post-hepatectomy when the majority of the hepatocytes were undergoing proliferation and started returning to the resting state level around 72 h when cellular proliferation declined.^23^ Accordingly, the hepatic BA levels were shown large increase at 38 h after PH and reduced at 72 h (Figure 1B). In two widely used mouse models for liver fibrosis, the reduced miR-122 levels were associated with the increased BA levels in the mouse fibrotic livers that were derived from the mice with bile duct ligation (BDL) or treatment of carbon tetrachloride (CCl_4_) (Figures 1C and 1D). Further analysis in a mouse model with non-alcoholic fatty liver disease (NAFLD) confirmed that the decreased hepatic miR-122 level was associated with the elevated serum BA levels in the mice fed with a high-fat diet (HFD) for 24 weeks (Figure 1E). Interestingly, *ex vivo* cell culture illustrated that the concentrations of secreted TBA were much higher in human HCC cell lines with lower miR-122 levels, like SNU449 and HepG2, compared with the cell line with higher miR-122 expression, like mouse hepatocyte cell line AML12 and HCC cell line Huh-7 (Figure S1).

Collectively, these data show that the reduced miR-122 expression is associated with elevated hepatic BA levels in numerous physiological and pathological processes of liver, which strongly suggests miR-122 may be involved in modulating BA metabolism.

### Liver-specific miR-122 loss of function results in an elevated hepatic BA level and altered BA spectrum

**Figure 2:**
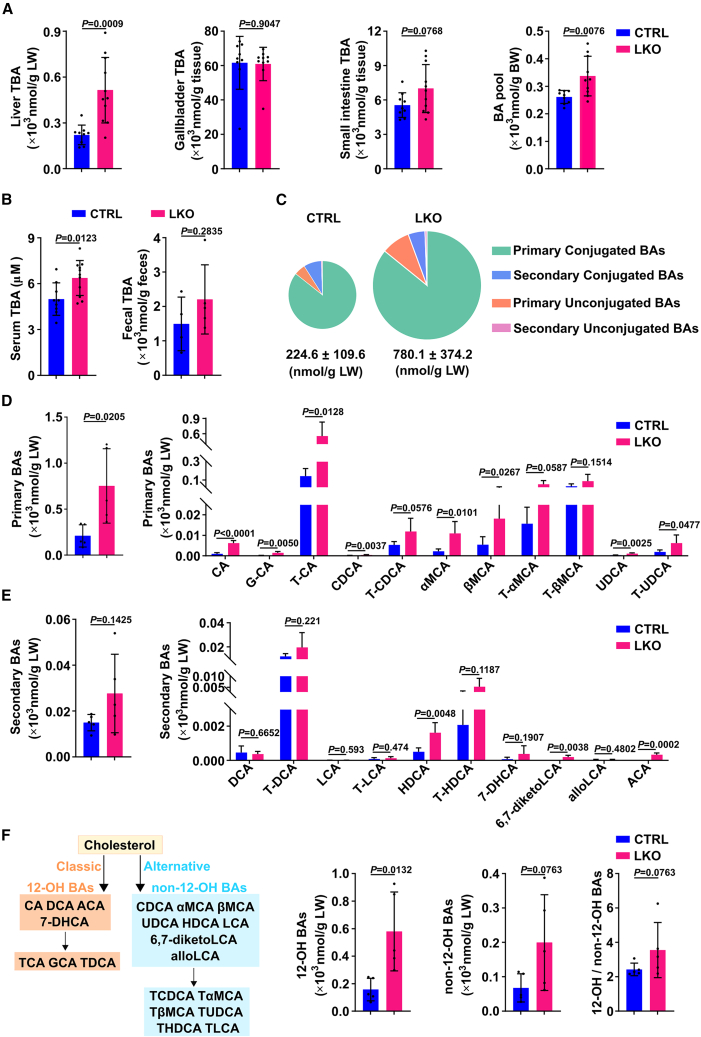
Liver-specific miR-122 loss of function results in an elevated hepatic BA level and altered BA spectrum (A) Elevated hepatic TBA and larger BA pool were observed in LKO mice. TBA in individual organs/tissues throughout the enterohepatic circulation were determined in CTRL and LKO mice, and total amounts of BA pool were obtained by combination of the values in liver, gallbladder, and small intestine. (B) Serum TBA was upregulated in LKO mice. Total amounts of TBA in feces and concentration of BAs in serum were determined in CTRL and LKO mice. (C) Loss of miR-122 dramatically increased hepatic BAs and altered BA spectrum. (D, E) The amounts of hepatic primary BAs were generally elevated (D), and the amounts of secondary BAs were mostly unchanged (E) in LKO livers. (F) Both 12-OH BAs and non-12-OH BAs were upregulated in LKO livers, with no change of the ratio of 12-OH/non-12-OH BAs. (Left) Classical and alternative pathways of BAs. For (B–F), fecal and hepatic BA composition of CTRL and LKO mice were determined by UHPLC-MS/MS. For (A–F), n = 5–10 of 8-week-old male mice per group, and the amounts of BAs were normalized to tissue weight or body weight (BW) in each mouse. The data from at least three independent experiments are presented as mean ± SD; *p* values were assessed by unpaired Student’s t test.

In an attempt to explore the intrinsic regulatory role of miR-122 in BA metabolism, *Mir122* liver-specific knockout mice (*Mir122*^*loxP/loxP*^ *Alb-Cre*^*+/−*^, named LKO) were generated (Figure S2A). LKO mice were born in a normal Mendelian ratio with no discernible defects at weaning and had normal fertility. DNA sequencing, Northern blotting, and quantitative real-time PCR (qPCR) were used to confirm the deletion of the *Mir122* gene and the loss of miR-122 expression in the hepatocytes of 8-week-old LKO mice (Figures S2B−S2D). There was no notable difference in body weight and liver weight (Figures S2E and S2F) between LKO mice and their littermate control mice (*Mir122*^*loxP/loxP*^ *Alb-Cre*^−/−^, named CTRL). Strikingly, analysis of BAs throughout the enterohepatic system of young LKO and CTRL mice (8-week-old) revealed that liver-specific miR-122 loss of function resulted in significant increased BA levels in the liver and serum but no significant changes in gallbladder, small intestine, and feces (Figures 2A and 2B). As a result, the BA pool size in LKO mice was increased by almost 29%, which was clearly mainly due to the elevated level of hepatic BAs in the LKO mice (Figure 2A).

To assess how the overall BA composition of the liver was affected by miR-122, we performed UHPLC-MS/MS to detect the profile of hepatic BAs in LKO mice and their control littermates. As expected, *Mir122* depletion dramatically increased BAs in mouse liver, which increased from 224.6 ± 109.6 nmol/g LW in CTRL mice to 780.1 ± 374.2 nmol/g LW in LKO mice (*p* < 0.05) (Figure 2C). Detailed analysis of each BA species showed that liver-specific miR-122 loss of function resulted in altered BA spectrum. The amounts of primary BAs were generally increased in LKO mice, and among them the most significant increases were CA, G-CA, T-CA, CDCA, αMCA, βMCA, UDCA, and T-UDCA (Figure 2D). On the other hand, the amounts of secondary BAs did not receive a significant upregulation in LKO livers, except for HDCA, 6,7-diketoLCA, and ACA (Figure 2E). Accordingly, the proportion of primary BAs in TBA increased markedly in LKO mice, whereas the proportion of secondary BAs reduced dramatically (Figure S2G), suggesting that *Mir122* knockout may promote liver BA synthesis.

BAs are synthesized in the liver via two different routes: the classical pathway to synthesize 12α-hydroxylated BAs (12-OH BAs) and the alternative pathway to produce non-12-OH BAs (Figure 2F, left panel). We found that both 12-OH BAs and non-12-OH BAs were significantly upregulated in LKO livers, and the ratio of 12-OH to non-12-OH BAs was not remarkably different between LKO and CTRL mice (Figure 2F). It is suggested that knockout of *Mir122* does not affect the preference of BA synthesis, and miR-122 may act on the common downstream of classical and alternative pathways for BA synthesis.

Taken together, these findings indicate miR-122 may play a suppressive role in hepatic BA synthesis.

### MiR-122 attenuates BA production via targeting BA synthesis gene *Hsd3b7*

**Figure 3:**
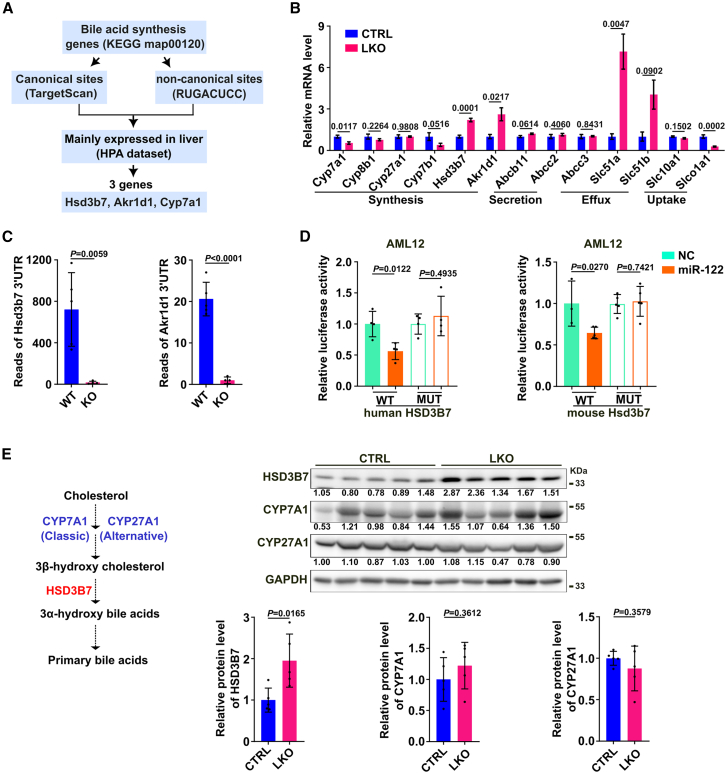
miR-122 inhibits HSD3B7 expression by directly binding to its 3′UTR (A) The screening workflow for potential miR-122 targets during BA synthesis. (B) Genes related to BA metabolism were examined in the livers of LKO and CTRL mice by qPCR (n = 3–6 mice per group). Gapdh was used as the internal control. The mean level in the CTRL group was set as 1. (C) The binding abilities of AGO protein to 3′UTRs of Hsd3b7 and Akr1d1 were significantly attenuated after the depletion of *Mir122*. AGO-CLIP-seq data were from Gene Expression Omnibus (GEO) datasets (GSE97058). (D) The luciferase activities of the reporters containing the wild-type HSD3B7 3′UTR from both human and mouse, but not those of the mutant HSD3B7-3′UTRs, were inhibited by miR-122 overexpression. (E) The protein level of HSD3B7 was significantly upregulated in LKO livers, whereas the protein levels of CYP7A1 and CYP27A1 remained unchanged. (Left) Schematic showing the reaction catalyzed by HSD3B7 in BA synthesis pathway. The protein levels were detected in the liver tissues from 8-week-old male CTRL or LKO mice (*n* = 5 mice per group). The level of target protein relative to GAPDH is indicated under each band and shown as histograms. For (C–E), the data from at least three independent experiments are presented as mean ± SD; *p* values were determined by unpaired Student’s t test.

To explore the mechanisms underlying miR-122-suppressed BA synthesis, we analyzed genes involved in primary BAs biosynthesis (KEGG map00120) to predict whether they contain canonical miR-122 target sequences (screened by TargetScan database) or non-canonical G-bulged motifs related to miR-122 through alignment with the RUGACUCC sequence.^24^ Hsd3b7, Akr1d1, and Cyp7a1, which mainly expressed in the liver, stood out as attractive candidates (Figure 3A). Then the expression of these candidates and other key genes in BA metabolism was examined in the livers of LKO mice and CTRL mice. Strikingly, more than 2-fold increases in the RNA levels of Hsd3b7 and Akr1d1 were observed in LKO mice compared with their control littermates, whereas Cyp7a1 was significantly downregulated (Figure 3B). Therefore, Hsd3b7 and Akr1d1 were selected for further validation. Subsequent analysis using AGO-CLIP-seq data from GSE97058 showed that the binding abilities of AGO protein to 3′UTRs of Hsd3b7 and Akr1d1 were both significantly impaired after *Mir122* knockout (Figure 3C), further suggesting that the 3′UTRs of mouse Hsd3b7 and Akr1d1 may have potential sequences to pair with miR-122. Furthermore, we were able to map miR-122-related but non-canonical G-bulged motifs in the 3′UTRs of human HSD3B7 and mouse Hsd3b7 and one canonical site in the 3′UTR of mouse Akr1d1 but not human AKR1D1 (Figure S3A). Dual-luciferase reporter analysis showed that overexpression of miR-122 significantly suppressed the *Firefly* luciferase activity of the reporters containing wild-type 3′UTRs of human HSD3B7 and mouse Hsd3b7 but not that of the mutant HSD3B7-3′UTRs and mouse wild-type Akr1d1-3′UTR reporter in both AML12 and HEK293T (Figures 3D, S3B, and S3C), indicating that miR-122 may directly suppress human HSD3B7 and mouse Hsd3b7 expression through their binding sequences at the 3′UTRs. The *Hsd3b7*, which encodes a BA synthesis enzyme that catalyzes two reactions required for inversion of 3β-hydroxy cholesterol to the 3α-hydroxy BAs, is a common downstream of classical and alternative pathways of BA synthesis (Figure 3E, left panel). Accordingly, the protein level of HSD3B7, but not two key synthesis enzymes CYP7A1 and CYP27A1, was significantly higher in LKO livers than in CTRL livers (Figures 3E and S3D).

**Figure 4:**
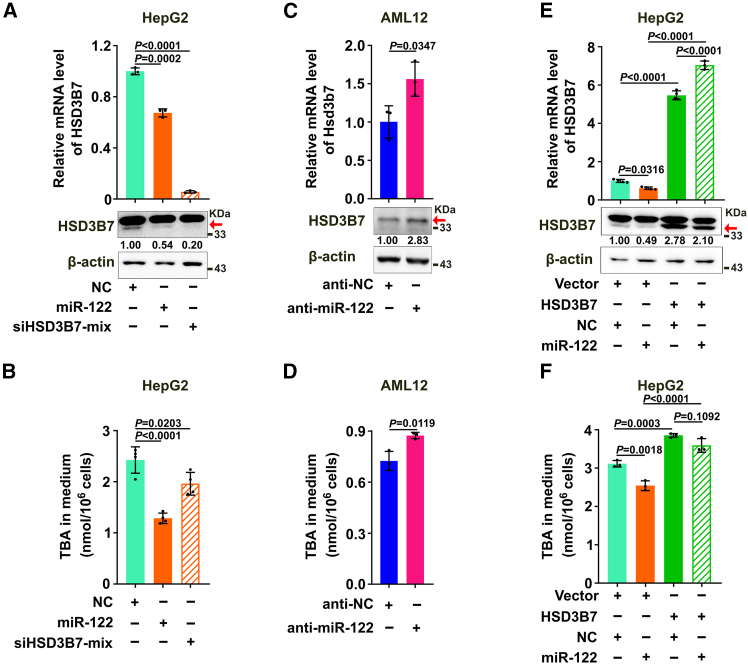
HSD3B7 is involved in miR-122-regulated reduction in BA production (A) miR-122 and siHSD3B7 repressed endogenous HSD3B7 expression. (B) Overexpression of miR-122 and knockdown of HSD3B7 decreased the production of BAs. (C) Suppression of endogenous miR-122 promoted the expression of HSD3B7. (D) miR-122 inhibitor increased the secretion of BAs. HepG2 or AML12 cells were transfected with RNA duplex or miRNA inhibitors. For (A) and (C), 48 h after transfection, cells were subjected to qPCR and western blotting analysis. For (B and D), 24 h after transfection, the fresh medium was changed, and then cells were cultured for another 48 h before the supernatants were collected for analyzing the concentration of TBA by biochemical analyzer. siHSD3B7-mix, a mixture of two siRNAs of HSD3B7. (E, F) Ectopic expression of HSD3B7 antagonized the effect of miR-122 in decreasing HSD3B7 expression (E) and the production of BAs (F). HepG2-HSD3B7 and HepG2-Vector sublines transfected with either NC or miR-122 mimics for 48 h were subjected to qPCR and western blotting (E). HepG2 stable cells were transfected with indicated RNA duplex for 24 h, and then the medium was changed freshly and cells were cultured for another 48 h before BA detection (F). “+” or “−”, presence (+) or absence (−) of the treatment. Red arrow indicates the band of HSD3B7 protein. For (A), (C), and (E), the protein level of HSD3B7 relative to β-actin is indicated under each band. Data are expressed as the mean ± SD of at least three independent experiments; *p* values were assessed by one-way ANOVA (A, B, E, and F) or unpaired Student’s t test (C and D).

To explore the role of HSD3B7 in miR-122-regulated BA synthesis, gain-of-function study was applied in HepG2 cell line with lower miR-122 level, and loss-of-function investigation was applied in normal hepatocyte cell line AML12 with higher miR-122 expression (Figure S1). Remarkably, overexpression of miR-122 reduced both mRNA and protein levels of HSD3B7 and led to less TBA production, which phenocopied the outcome of siHSD3B7 (Figures 4A and 4B). Antagonism of endogenous miR-122 by anti-miR-122 not only significantly enhanced the cellular RNA and protein levels of HSD3B7 but also induced TBA levels in AML12 (Figures 4C and 4D). Notably, ectopic expression of HSD3B7 rescued miR-122-induced reduction in BA production (Figures 4E and 4F).

These findings imply that in hepatocytes miR-122 may inhibit BA production by suppressing BA synthesis enzyme HSD3B7.

### BA sequestrant alleviates liver tumor growth induced by miR-122 deficiency in hepatocytes, whereas intrahepatic HSD3B7 overexpression reverses miR-122-reduced tumor burden

**Figure 5:**
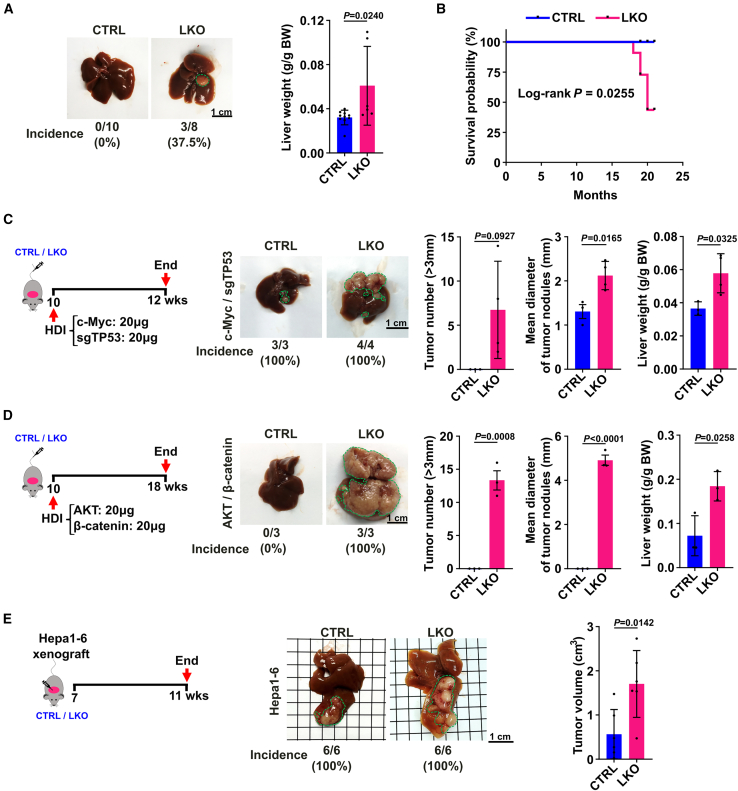
*Mir122* depletion in hepatocytes promotes the growth of oncogene-driven liver cancer and orthotopic liver xenografted tumors in mice (A) Conditional knockout of *Mir122* in hepatocytes promoted the development of liver tumor with age (n = 8–10 per group). (Left) Representative photographs of livers from CTRL and LKO male mice at the age of 20 months. (Right) The ratio of liver weight to body weight. (B) The survival time of male LKO mice was shorter than CTRL mice (n = 8–11 mice per group). (C, D) *Mir122* depletion in hepatocytes significantly increased tumor burden in oncogene-driven HCC mouse models (n = 3–4 mice per group). (E) miR-122 deficiency in hepatocytes significantly promoted liver tumor growth in orthotopic xenograft model (*n* = 6 mice per group). For (C–E), left, cartoon depicting the establishment of mouse liver tumor model; middle, representative images of livers with tumor; right, quantification of tumor number (>3 mm), mean diameter of tumor nodules as well as liver/body weight (C, D) or tumor volume (E) in CTRL and LKO mice. For (A, left) and (C–E, middle), the numbers and proportion below indicate the tumor incidence, and tumor nodules were highlighted by green dashed lines. Scale bars, 1 cm. Data are presented as mean ± SD; *p* values were examined either by unpaired Student’s t test (A, C–E, right) or by Log rank test (B).

Known as a tumor suppressor gene, miR-122 has been reported to play an important role in the development of HCC. In line with previous reports,^19^^,^^21^ over time our *Mir122* LKO mice gradually developed phenotypes of liver injury, inflammation, and fibrosis, as evidenced by higher level of serum AKP at 12 weeks old (Figure S4A), upregulation of pro-inflammation factors such as interleukin-6 (Il-6) and Ccl2 at 33 weeks old (Figure S4B), and accumulation of activated hepatic stellate cells and their product collagen that were determined by α-SMA and Sirius Red staining at 33 weeks old (Figures S4C–S4E), and eventually spontaneously developed liver tumor with age. At around 20 months of age, approximately 37.5% of male LKO mice developed HCC (Figure 5A), and the survival of male LKO mice was significantly shortened compared to CTRL littermates (Figure 5B).

Then an important question arises as to whether the increase of BAs in liver parenchyma of *Mir122* LKO mice in the early stage is associated with the development of HCC. To solve this point, a well-established transposon-based mouse autochthonous model of liver cancer, in which intrahepatic delivery of oncogenes, such as a combination of c-Myc, Cas9, and a single guide RNA targeting TP53 (c-Myc/sgTP53) or a combination of myr-AKT and β-catenin (AKT/β-catenin), by hydrodynamic tail vein injection (HDI) that leads to the initiation of liver cancers, was first applied in LKO and CTRL mice. Strikingly, we observed rapid outgrowth of multifocal c-Myc/sgTP53-induced and AKT/β-catenin-induced liver tumors in LKO mice, showed by significantly elevated tumor number, tumor size, and liver/body weight ratio and increased tumor incidence (Figures 5C and 5D), compared with CTRL mice, suggesting that miR-122 deficiency in hepatocytes synergizes with oncogene activation to promote HCC development. Moreover, we employed an orthotopic liver xenograft model to gain new insights into the role of miR-122 in liver parenchymal cells of tumor microenvironment in HCC progression. Compared with the Hepa1-6 xenografts from the CTRL group, the xenografts from the LKO mice displayed a dramatic increase in growth (Figure 5E). These findings suggest that *Mir122* depletion in hepatocytes in paracancerous tissues may promote HCC tumor growth.

**Figure 6:**
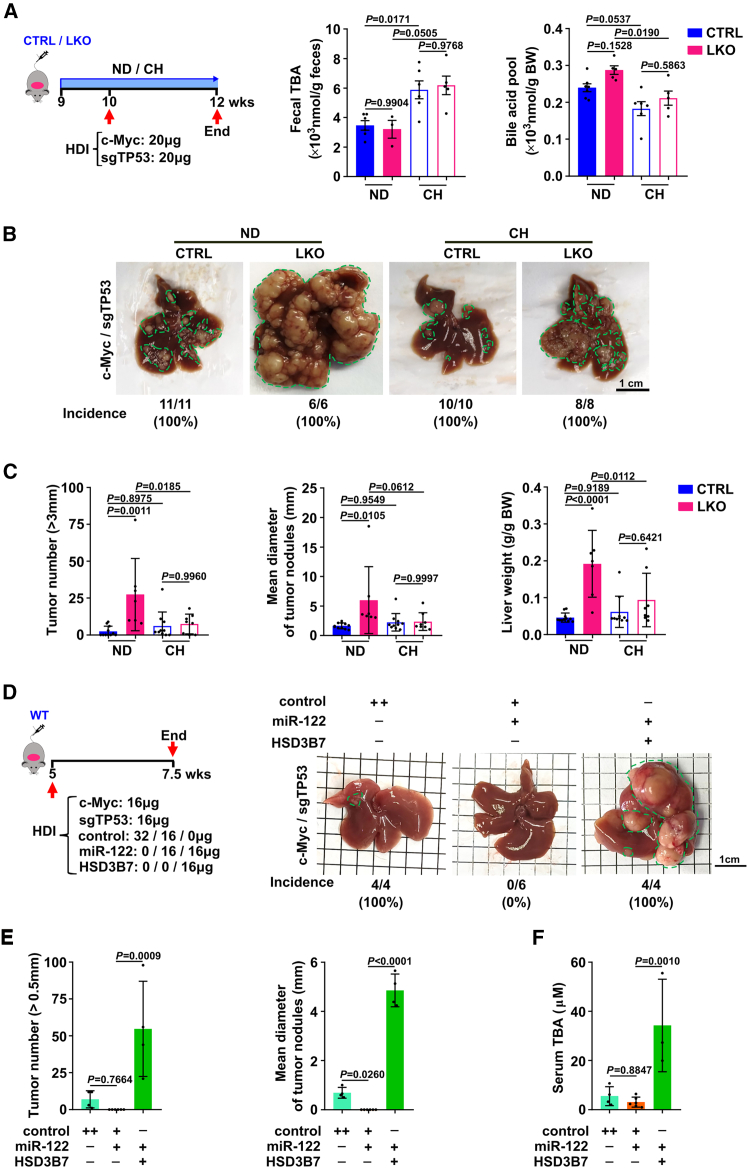
BA sequestrant alleviates liver tumor growth induced by miR-122 deficiency in hepatocytes, whereas intrahepatic HSD3B7 overexpression reverses miR-122-mediated reduction in tumor burden (A) Cholestyramine treatment dramatically increased fecal TBA and decreased BA pool in CTRL and LKO mice (n = 3–7 per group). (Left) Experimental design of cholestyramine treatment in mice upon HDI of c-Myc/sgTP53. Nine-week-old CTRL and LKO mice receiving a normal diet (ND) or 2% (w/w) cholestyramine (CH) diet for 1 week, then BA levels in feces and BA pool were measured. (B, C) *Mir122* depletion resulting in tumor burden increase was abrogated by cholestyramine treatment (n = 6–11 mice per group). CTRL and LKO mice were pretreated with ND or CH diet for 1 week, followed by HDI of c-Myc/sgTP53, and then harvested after 2 weeks. For (B), the representative liver images; for (C), quantification of tumor number (>3 mm) (left), mean diameter of tumor nodules (middle), and liver/body weight ratio (right) of mice were presented. (D–F) The tumor-suppressed effect induced by intrahepatic delivery of miR-122 was blocked by the forced expression of HSD3B7 in hepatocytes (n = 4–6 mice per group). For (D), left, cartoon depicting the working flow of intrahepatic delivery of miR-122 or HSD3B7 via HDI; right, the representative photographs of livers with tumors. For (E), left, tumor number (>0.5 mm); right, the mean diameter of tumor nodules. For (F), the level of serum BAs was determined enzymatically. For (B) and (D), the numbers and proportion below indicate the tumor incidence, and tumor nodules were highlighted by green dashed lines. Scale bars, 1 cm (B and D). “+” or “−”, presence (+) or absence (−) of the treatment. The data from at least three independent experiments are presented as mean ± SD; *p* values were measured by one-way ANOVA (A, C, E, and F).

In order to investigate the role of the increase of BA production caused by miR-122 deficiency in liver parenchyma on HCC development, a BA sequestrant cholestyramine was introduced to LKO and CTRL mice upon HDI of c-Myc/sgTP53 (Figure 6A, left panel). As expected, cholestyramine treatment dramatically enhanced the excretion of BAs to reduce *in vivo* BA pool (Figure 6A). Interestingly, compared with CTRL mice, liver tumor burden from LKO mice upon HDI of c-Myc/sgTP53 was increased, but this promotive effect was almost completely abolished when administered with cholestyramine (Figures 6B and 6C). To further confirm that HSD3B7 was a culprit in the development of HCC induced by hepatocyte-specific knocking out of *Mir122*, a combination of c-Myc/sgTP53, with or without miR-122 and HSD3B7, was delivered into hepatocytes in *C57BL/6J* mice livers via HDI of the transposon vectors (Figures 6D and S5). Intrahepatic ectopic expression of miR-122 dramatically reduced tumor incidence (Figure 6D, control vs. miR-122: 100% vs. 0%) and tumor growth (Figure 6E, bar1 vs. bar2) in c-Myc/sgTP53-induced HCC model. Strikingly, overexpression of HSD3B7 in hepatocytes rescued the miR122-reduced liver tumor burden, showed by significantly elevated tumor incidence (Figure 6D, miR-122 vs. miR-122+HSD3B7: 0% vs. 100%) and induced tumor number and size (Figure 6E, bar2 vs. bar3). Consistently, the decrease of serum BA levels induced by miR-122 was remarkably abolished by intrahepatic ectopic expression of HSD3B7 in mice upon c-Myc/sgTP53-HDI (Figure 6F). Collectively, these data suggest that the induction of BAs caused by the forced expression of HSD3B7, which is due to miR-122 deficiency in paracancerous tissues, may promote occurrence and development of HCC.

**Figure 7:**
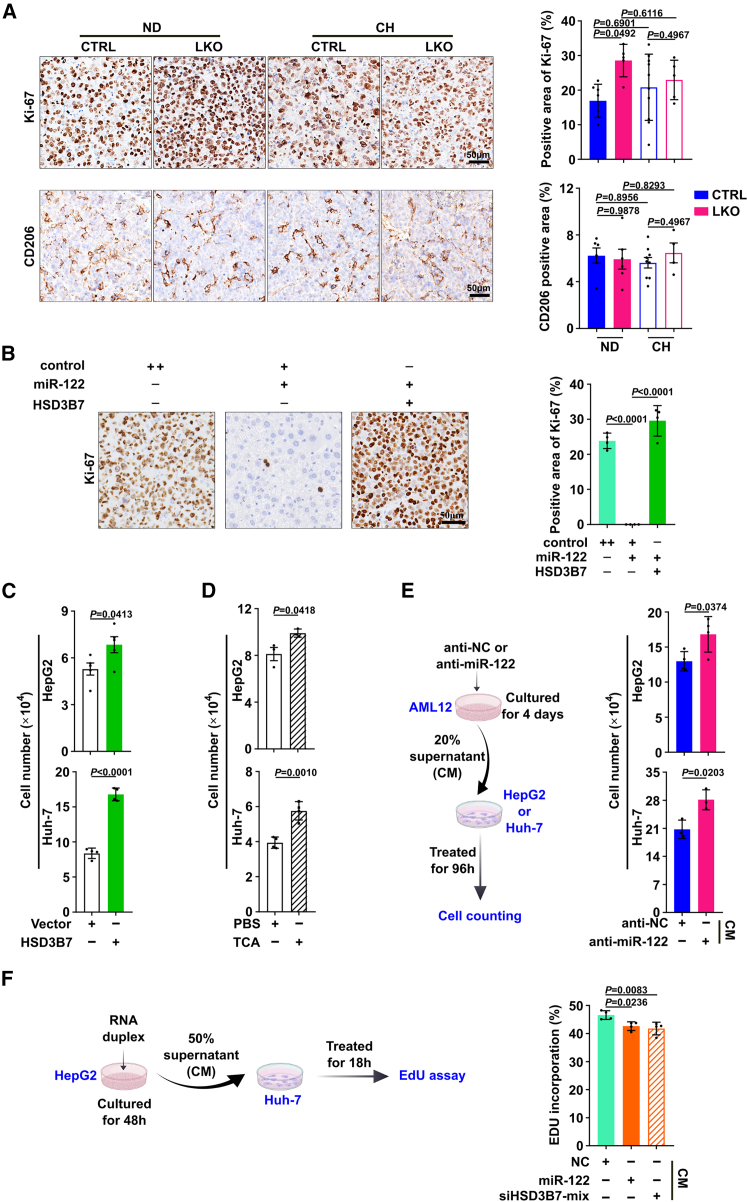
Elevated BA levels driven by miR-122 deficiency in hepatocytes promote tumor cell proliferation (A) The Ki-67 signals were increased after the deletion of *Mir122* in hepatocytes but were abolished by BA sequestrant. Representative images of IHC staining with anti-Ki-67 (upper) or anti-CD206 (lower) antibody and quantification of positive area (right) by ImageScope were shown. (B) The decrease of Ki-67 signals upon intrahepatic delivery of miR-122 was abolished by the forced expression HSD3B7 in liver in c-Myc/sgTP53-induced HCC model. Scale bar, 50 μm. (C) Ectopic expression of HSD3B7 promoted the tumor cell growth. (D) The growth of HCC cells was accelerated by the treatment of TCA. (E) The growth of tumor cells was facilitated after administration of supernatants from AML12 transfected with anti-miR-122. (Left) Working flow of the cell counting assay. (F) DNA replication of cancer cells was diminished by the supernatants from cells overexpressing miR-122 or siHSD3B7. (Left) Working flow of the EdU incorporation assay. “+” or “−”, presence (+) or absence (−) of the treatment. The data from at least three independent experiments are presented as mean ± SD; *p* values were measured by one-way ANOVA (A, B, and F, right) or unpaired Student’s t test (C−E).

Emerging evidence suggests that the abnormal accumulation of BAs either acts as hepatomitogens to facilitate tumor proliferation^15^ or acts as cell-signaling mediators to promote M2 macrophage polarization to create immunosuppressive tumor microenvironment favorable for the growth of HCC cells.^16^ To this end, we observed that cholestyramine treatment abrogated the stimulatory effect of *Mir122* depletion in hepatocytes on cancer cell proliferation, which was determined by Ki-67 staining (Figure 7A). However, the amounts of M2 polarized macrophages detected by CD206 staining were similar among LKO and CTRL mice without or with cholestyramine treatment (Figure 7A). Consistently, ectopic expression of HSD3B7 in hepatocytes abolished the inhibitory effect of intrahepatic delivery of miR-122 on cancer cell proliferation in c-Myc/sgTP53-induced HCC model (Figure 7B). Moreover, overexpression of HSD3B7, or administration of the most abundant primary BA TCA, or treatment with the supernatants from AML12 after blocking endogenous miR-122, significantly promoted tumor cell growth (Figures 7C–7E and S6), whereas the DNA replication of cancer cells was diminished by the supernatants from cells overexpressing miR-122 or siHSD3B7 (Figure 7F). These results indicate the induction of BAs caused by the decrease of miR-122 in hepatocytes may facilitate HCC tumor cell proliferation.

### Dysregulation of miR-122-HSD3B7-BA pathway in paracancerous tissues during human HCC development

**Figure 8:**
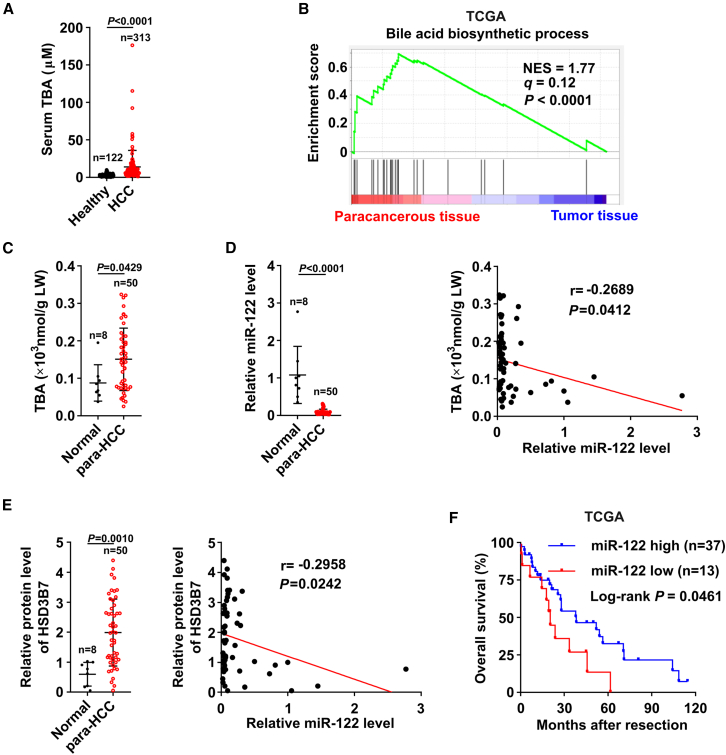
Dysregulation of miR-122-HSD3B7-BAs signaling during human HCC development (A) TBA level of serum samples was higher in patients with HCC (*n* = 122) than that in the healthy people (*n* = 313). (B) The BA biosynthetic process was enriched in the paracancerous tissues from HCC patients. *q* and *p* values were determined by GSEA, and data were from TCGA dataset. (C) TBA level was higher in the paracancerous tissues compared with normal liver tissues. (D) Decreased miR-122 expression in the paracancerous tissues was associated with elevated TBA. (E) Upregulation of HSD3B7 was associated with the downregulation of miR-122 in para-HCC. GAPDH was used as an internal control. For (C–E), TBA and the expression of miR-122 and HSD3B7 were assessed in 50 paracancerous tissues from HCC patients (para-HCC) and in 8 normal liver tissues from patients undergoing resection of hepatic hemangiomas (Normal). For (E), HSD3B7 was detected by immunoblotting, as presented in Figure S8. (F) A Kaplan-Meier plot revealed the association between a lower expression of miR-122 in paracancerous tissues from HCC patients and a shorter overall survival. Based on the minimum *p* value approach, the 74^th^ percentile of the miR-122 level in 50 paracancerous tissues of HCC was chosen as the cut-off value for separating the miR-122 high-level group (*n* = 37) from the miR-122 low-level group (*n* = 13). Data were from TCGA. *p* values were determined by unpaired Student’s t test (A, C, D, and E, left), Pearson correlation analysis (D and E, right), or Log rank test (F). Data are shown as mean ± SD (A, C, D, and E, left).

We further validated the function of miR-122-HSD3B7-BAs axis in human HCC samples. As shown, the serum TBA concentrations from HCC patients were significantly increased compared with those from healthy people (Figure 8A), suggesting that the upregulation of BA metabolism may play an important role during the occurrence and development of HCC. Notably, GSEA analyses on the transcriptome profiles of 50 paired HCC tumor tissues and their matched paracancerous tissues (liver parenchymal tissues that have not yet become cancerous but have developed lesion) from TCGA database disclosed that genes involved in BA biosynthetic process were enriched in the paracancerous tissues but not tumor tissues (Figure 8B), indicating that the robust induction of BA synthesis in the paracancerous tissues may be responsible for the elevated BAs in HCC patients. Consistently, in our study cohort, the TBA level was significantly higher in the paracancerous tissues, but not so dramatic induction in HCC tumor tissues, compared with normal liver tissues from patients undergoing resection of hepatic hemangiomas (Figures 8C and S7). Interestingly, the level of miR-122, remarkably lower in the paracancerous tissues compared with the normal liver tissues, was negatively associated with TBA level (r = −0.2689, *p* = 0.0412) (Figure 8D). Consistently, the significant upregulation of HSD3B7 was observed in 92% (46 of 50) of the paracancerous tissues from HCC patients, which was associated with downregulation of miR-122 (r = −0.2958, *p* = 0.0242) (Figures 8E and S8). Moreover, the Kaplan-Meier survival analysis revealed a lower miR-122 level from paracancerous tissues was significantly associated with a worse overall survival for HCC patients (TCGA dataset) (Figure 8F). Our results indicate that the enhancement of BA metabolism resulting from the downregulation of miR-122 in paracancerous tissues could be a common event and likely a risk factor in human HCC.

In summary, we disclose that the deficiency of miR-122 in liver parenchymal cells (hepatocytes) of the paracancerous tissues leads to the increase of BA production by accelerating the expression of its target gene *HSD3B7*, thereby facilitating HCC tumor cell growth.

## Discussion

miR-122, a mammalian liver-specific miRNA, is involved in various metabolism processes in liver, such as the metabolism of lipid,^19^^,^^25^ glucose,^26^ and iron.^27^ Nonetheless, whether metabolism of BAs, the important liver product, can be regulated by miR-122 is still obscure. Herein, we find a novel biological function of miR-122 in attenuating *de novo* BA production in liver via targeting BA synthesis gene *HSD3B7*, and the loss of miR-122 in hepatocytes in paracancerous tissues leads to enhanced BA biosynthesis, which in turn facilitates cancer cell proliferation and HCC tumor growth.

Despite the extensive characterization of the functions of BAs in orchestrating whole-body metabolism and leading to numerous diseases,^28^ multiple aspects of how BA pool are tightly maintained remain unclear. Liver-specific knockout of BA-sensing receptor FXR, which was once thought to be a major regulator of BA homeostasis, only slightly increased the size of the BA pool but not hepatic BAs.^8^^,^^9^ Thus, as the primary organ of BA synthesis, the liver is likely to have evolved another system to carefully control BA synthesis. Previous studies have reported that the levels of total cholesterol, a precursor for BA synthesis, were reduced upon the absence of miR-122 in mice, which indicated that miR-122 might act on the metabolism of BA.^19^^,^^25^ Intriguingly, unlike FXR that was elicited by BAs, we disclosed that liver-specific knockout of *Mir122* significantly increased BA abundance in liver and altered BA composition by directly upregulating BA synthesis enzyme HSD3B7. Moreover, reduced miR-122 expression was associated with elevated hepatic BA levels in liver regeneration, liver fibrosis, and fatty liver disease, implicating that BA metabolism modulated by miR-122 might play a pivotal role in a variety of physiological and pathological processes of liver. Surprisingly, *Mir122*-depletion-induced BA production resulted in the decreased Cyp7a1 mRNA level (Figure 3B), which might be in an FXR-dependent negative-feedback manner, whereas a previous study demonstrated that miR-122 could be upregulated by FXR,^29^ indicating a complex crosstalk between miR-122-regulated BA production and FXR-coordinated BA homeostasis. These might ensure that BAs are modulable in various physiological and pathological phenomena to avoid BA accumulation and toxicity. These findings extend our understanding on the regulatory network of BA homeostasis.

Although silencing of HSD3B7 led to less TBA production, phenocopying the outcome of miR-122 expression and the rescue assay showed that overexpression of HSD3B7 could almost rescue the reduction of BA induced by miR-122 overexpressing, suggesting that miR-122 may inhibit BA production by suppressing HSD3B7. We noticed that compared with miR-122 overexpression, inhibition of HSD3B7 caused a slightly less extent of TBA reduction (Figure 4B), implying that miR-122 may have some other ways to repress BA production in addition to targeting HSD3B7. Two previous studies have reported that miR-122 inhibited CYP7A1 mRNA level by targeting its 3′UTR.^30^^,^^31^ However, compared with their control littermates, mRNA of Cyp7a1 was significantly downregulated (Figure 3B), which was consistent with the previous finding that miR-122 induced CYP7A1 mRNA level by targeting GUTL1,^32^ and protein of Cyp7a1 was not changed in LKO mice livers (Figures 3E and S3D), implying that Cyp7a1 might not be a target of miR-122 in our experimental setting. The regulatory network of miR-122 on BA metabolism appears to be complex and may be highly dependent on the cellular context. Further research is warranted to explore other mechanisms by which miR-122 repress BA production.

HSD3B7, predominantly expressed in the liver, is a membrane-bound enzyme of the endoplasmic reticulum that transforms the steroid into primary BAs. There are few studies on HSD3B7, and most of them focus on patients with mutations of HSD3B7 developing a congenital BAs synthesis defect (CASD), which leads to a progressive cholestatic liver disease that is clinically responsive to primary BAs treatment.^33^ Only one study disclosed that loss of *Hsd3b7* in mice led to a complete lack of primary BAs and the accumulation of 3β,7α-dihydroxy- and 3β,7α,12α-trihydroxy-Δ(5)-cholanoic acids, the unactive forms of BAs, thereby resulting in vitamin deficiency and cholesterol malabsorption.^34^ However, how HSD3B7 is tightly controlled and whether its dysfunction contributes to the development of HCC is still obscure. In this study, we verified that miR-122 directly bound with the non-canonical G-bulged motif at the HSD3B7 3′UTR to silence its expression and demonstrated that the upregulation of HSD3B7 was responsible for miR-122-deficiency-induced increase of BA levels. Furthermore, we discovered that the induction of BAs caused by the forced expression of HSD3B7 promoted cancer cell growth and proliferation and defined that HSD3B7 was frequently upregulated in the paracancerous tissues of HCC patients, suggesting the potential oncogenic activity of HSD3B7 and its potential application in cancer therapy, which was in accordance with the recent study about the essential function of HSD3B7 in ccRCC cell survival.^35^

HCC is the malignant tumor with metabolic reprogramming.^36^ However, the role of BA metabolism in the occurrence and progression of HCC remains controversial. On the one hand, elevated hepatic BAs may lead to a poor prognosis in HCC patients.^11^ On the other hand, HCC cancer cells might lose their original liver-specific metabolic function during hepatocarcinogenesis, which is manifested by the downregulation of the most key proteins of BA metabolism.^12^ Moreover, it has been shown that BAs can stimulate anti-tumor immunity by activating the hepatic NKT cells,^13^ but it also promotes hepatocarcinogenesis by accumulating M2-like tumor-associated macrophages,^16^ or by provoking senescence-related secretory phenotype of hepatic stellate cells,^14^ or by inducing tumor cell growth.^15^ Herein, we found that BA levels were elevated in mouse fibrotic liver and non-alcoholic fatty liver, two of the most common precancerous lesions of HCC. Interestingly, elevated serum BA levels in HCC patients were mainly due to robust induction of BA synthesis in the paracancerous tissues (usually defined as precancerous lesions of HCC), as evidenced by the enrichment of BA biosynthetic process and the induction of BAs in the paracancerous tissues rather than in HCC tumor tissues. Moreover, *in vivo* study showed that BA depletion by cholestyramine abrogated *Mir122* LKO-driven liver tumor growth and cancer cell proliferation. Consistently, the inhibitory effect induced by overexpression of miR-122 in hepatocytes on c-Myc/sgTP53-driven tumor growth was completely blocked by forced expression of HSD3B7, indicating that the role of miR-122-HSD3B7-BA regulatory axis in paracancerous tissues on tumor growth. More importantly, *in vitro* study showed that supernatants from either miR-122 overexpression or HSD3B7 knockdown reduced the DNA replication of cancer cells, whereas HSD3B7 overexpression, administration of TCA, or supernatants from miR-122 antagonism promoted cancer cell growth, suggesting that abnormal accumulation of BAs by miR-122 deficiency may act as hepatomitogens to facilitate cancer cell proliferation. These findings are in accordance with the observation that hepatic BAs were induced and miR-122 was downregulated when the majority of hepatocytes were undergoing proliferation during liver regeneration (Figure 1B). Thus, our findings resolve the apparent paradox that HCC patients exhibit elevated hepatic and serum BA levels despite downregulated BA metabolism in tumor tissues. This study suggest that the enhancement of BAs synthesis in paracancerous tissues may have carcinogenic potential during the HCC progression. Furthermore, we propose two potential therapeutic strategies: (1) cholestyramine, an FDA-approved hyperlipidemia medication and (2) HSD3B7 knockdown could serve as viable treatment options for HCC patients with reduced miR-122 in paracancerous tissues.

It is well recognized that miR-122 acts as a tumor suppressor and decreased miR-122 levels in cancer cells have been associated with poor prognosis and excessive proliferation^22^ and metastasis^37^ in HCC. However, previous studies mainly focus on the functions of miR-122 in HCC tumor cells and rarely discuss the role of miR-122 in hepatocytes in the paracancerous tissues. In this study, we first found that compared with the normal liver tissues, the expression of miR-122 was significantly downregulated in the paracancerous tissues. The substantial difference of miR-122 between paracancerous liver tissues and normal controls may be attributed to the following reasons. First, the paracancerous liver tissues might be already under pathological stress or early stages of transformation like hepatitis and cirrhosis. It was reported that the reduced expression of miR-122 was observed in numerous pathological processes of liver, such as hepatitis virus infection,^38^ non-alcoholic^39^ and alcoholic fatty liver disease,^40^ or aflatoxin exposure,^22^ which were considered as precancerous lesions of HCC. Indeed, the transcription activator peroxisome proliferator-activated receptor gamma (PPARγ) suppressed by binding with HBX protein during HBV infection,^38^ the transcription regulator GRLH2 induced by chronic alcohol,^40^ and C/EBPα suppressed by aflatoxin exposure^22^ have been shown to lead to miR-122 reduction in hepatocytes. Second, the paracancerous liver tissues, although histologically non-cancerous, may still exhibit cytokines alterations due to their proximity to the tumor microenvironment. It was well known that transforming growth factor β (TGF-β) and interleukin-17, two cytokines that were frequently elevated during the development of HCC,^41^^,^^42^ suppressed the expression of miR-122,^43^^,^^44^ which might be another contributing factors to the downregulation of miR-122 in paracancerous liver tissues. Furthermore, we disclosed that in murine models of oncogene-driven HCC or Hepa1-6 xenografts, *Mir122* depletion in liver parenchymal cells of paracancerous tissues promoted tumor cell growth, but this promotive effect was almost reversed with cholestyramine. Increasing amounts of BAs were associated with the reduced expression of miR-122 in the paracancerous tissues from HCC patients. And lower expression of miR-122 from paracancerous tissues was linked to a worse prognosis for HCC patients. Taken together, our study first revealed that enhancement of BA metabolism by the reduced expression of miR-122 in paracancerous tissues could be a common occurrence in HCC, and potential future therapeutic strategies aiming at targeting BA metabolism or restoring miR-122 in hepatocytes in paracancerous tissues might be vital for HCC treatment.

In summary, we identify an miR-122-HSD3B7-BA regulatory axis and elucidate its function in tumor growth of HCC, which may be exploited for HCC treatment.

## Materials and methods

More details are provided in the supplemental information.

### Mouse model study

All mice were housed under specific pathogen-free conditions at the Sun Yat-Sen University Laboratory Animal Center. All procedures for animal experiments were performed in accordance with the Guide for the Care and Use of Laboratory Animals (National Institutes of Health publication nos. 80-23, revised 1996) and according to institutional ethical guidelines for animal experiments of Sun Yat-sen University (SYSU-IACUC-2023-000330).

For PH model, 8- to 12-week-old male *C57BL/6* mice were anesthetized with isoflurane, and two-thirds of the liver was surgically removed as previously described.^23^ Ligation and resection of the median lobe and the left lateral lobe were performed separately. Liver tissues were harvested at 0, 18, 24, 38, and 72 h after PH.

For mouse liver fibrosis models, male *C57BL/6* mice with 6 weeks of age were used. In the BDL model, the common bile duct was ligated for 21 days as previously described,^45^ and sham-operated mice were served as negative controls. In the model of chemical-induced liver fibrosis, mice were intraperitoneally injected with CCl_4_ (0.5 μg/g body weight, mixed with corn oil at 1:5) twice a week for 4 weeks. Corn oil-injected mice were used as negative controls.

For HFD model, 8-week-old male *C57BL/6* mice were randomly assigned to be fed over a 25-week period with either a standard diet or a high-fat diet (TP23520, Trophic Animal Feed High-tech Co. Ltd, Jiangsu, China). After fasting for 8 h, mice were sacrificed and harvested.

To construct *Mir122* liver-specific knockout mice, the *Mir122*^*loxP/loxP*^ mice in *C57BL/6J* background were generated by Nanjing Biomedical Research Institute of Nanjing University via CRISPR/Cas9 system. *Albumin-Cre* (*Alb-Cre*) mice were originally purchased from Shanghai Model Organisms Center Inc. The *Mir122*^*loxP/loxP*^ mice were crossed to *Alb-Cre* mice to generate *Mir122* liver-specific knockout mice (*Mir122*^*loxP/loxP*^ *Alb-Cre*^*+/−*^, named LKO), and their littermates *Mir122*^*loxP/loxP*^ *Alb-Cre*^−/−^ mice were used as controls (named CTRL).

For autochthonous liver tumor model, a plasmid mixture of 20 μg pT3-EF1aH-c-Myc (c-Myc)^46^ and 20 μg pX330-U6-sgTP53-CBh-hspCas9 (sgTP53),^47^ or a plasmid mixture of 20 μg pT3-EF1aH-myr-AKT (AKT)^46^ and 20 μg pT3-β-catenin (β-catenin),^47^ together with 1.6 μg transposase-encoding vector (pPGK-SB13, BioVector NTCC Inc.) that was dissolved in 2 mL of 0.9% NaCl buffer was hydrodynamically injected (HDI) into the tail veins of LKO and CTRL male mice within 10 s. After 2 weeks (for c-Myc/sgTP53) or 8 weeks (for AKT/β-catenin), mice were sacrificed for the examination of liver tumors. For forced expression of miR-122 and HSD3B7 in liver, the plasmids mixture of 16 μg pT3-EF1aH-c-Myc (c-Myc),^46^ 16 μg pX330-U6-sgTP53-CBh-hspCas9 (sgTP53),^47^ and 2 μg pPGK-SB13, with or without 16 μg of pT3-EF1aH-mmu-miR-122-precursor (miR-122), pT3-EF1aH-HSD3B7 (HSD3B7), and the matched control vector (pT3-EF1aH, named control), was dissolved in 0.9% NaCl buffer and then injected into the tail veins of wild-type mouse via HDI. pT3-EF1aH-c-Myc, pT3-EF1aH-myr-AKT, and pT3-EF1aH were gifts from Prof. Jun-Fang Ji (Zhejiang University, Hangzhou, China). pX330-U6-sgTP53-CBh-hspCas9 and pT3-β-catenin were gifts from Prof. Bin Zhao (Zhejiang University).

For xenograft model, 4 × 10^5^ Hepa1-6 cells were inoculated under the capsule of the left hepatic lobe of 7-week-old LKO and CTRL male mice. After 4 weeks, mice were sacrificed and tumor volume (V) was monitored by measuring the length (L) and width (W) with calipers and calculated with the formula: $V=L\times W^{2}\times 0.5$.

For cholestyramine treatment model, 9-week-old male LKO and CTRL mice were fed with chow diet or diet supplemented with 2% (w/w) cholestyramine resin (C4650, Sigma-Aldrich, St. Louis, MO, USA) for 1 or 3 weeks. Mice were fasted for 8 h before sacrificed, and the indicated tissues were determined.

### Cell lines

Human hepatoma cell lines (Huh-7 and SNU449), hepatoblastoma cell line (HepG2), mouse hepatocytes cell line (AML12), human embryonic kidney 293 cell line (HEK293T), and mouse hepatoma cell line (Hepa1-6) were cultured in Dulbecco’s modified Eagle’s medium (10-013-CVRC, Corning, New York, USA), supplemented with 10% fetal bovine serum (FBS) (086–150, WISENT, Canada) in a humidified atmosphere of 5% CO_2_ at 37°C. The HepG2 cell subline that stably expressed human HSD3B7 (HepG2-HSD3B7) and the matched control line (HepG2-Vector) were used in this study.

## Measurement of TBA

TBA levels in human and mouse tissues were measured enzymatically using Total Bile Acid Assay Kit (E003-2, Nanjing Jiancheng Bioengineering Institute). Serum samples from mice were measured directly according to the manufacturer’s instructions. For measurement of TBA in mouse liver, gallbladder, small intestine (with contents), and feces, and in human HCC tissues and paracancerous tissues, samples were minced and extracted in 70%–75% ethanol at 55°C for 4 h. The extracts were shaken, centrifuged, and TBA concentration of the supernatants was determined.

Ultra-high performance liquid chromatography-mass spectrometry/mass spectrometry (UHPLC-MS/MS) to detect the profile of hepatic BAs in mice was performed by BIOTREE (Shanghai, China). Specifically, ∼50 mg liver tissues of either five LKO male mice or five control littermates with 8 weeks of age were collected for detection. Finally, 22 types of BA in total were detected and listed in Table S1.

For determining secreted TBA from different cell lines, cells with indicated transfection were seeded in a 6-well plate for 48 h before medium was collected. The medium was centrifuged at 3,000 g for 10 min, and TBA from supernatants were measured by biochemical analyzer.

### Oligonucleotides and plasmids

All miRNA mimics (miR-122), small interfering RNA (siRNA), the negative control (NC), RNA for miRNA and siRNA, miR-122 inhibitor (anti-miR-122), and the negative control for miR-122 inhibitor (anti-NC) were purchased from RIBOBIO (Guangzhou, China). siRNA that targets the human HSD3B7 (GeneBank accession No. NM_001142778.2) transcript was designated as siHSD3B7. All oligonucleotide sequences are listed in Table S2.

The expression plasmids pGL3cm-human-HSD3B7-3′UTR-WT, pGL3cm-human-HSD3B7-3′UTR-MUT, pGL3cm-mouse-Hsd3b7-3′UTR-WT, pGL3cm-mouse-Hsd3b7-3′UTR-MUT, pGL3cm-mouse-Akr1d1-3′UTR-WT, pT3-EF1aH-mmu-miR-122-precusor, pT3-EF1aH-HSD3B7, and pCDH-HSD3B7 were generated as described in the supplemental materials and methods.

### Analysis of gene expression

Real-time quantitative polymerase chain reaction (qPCR) and Northern blotting were performed to evaluate RNA levels. Immunoblotting and immunohistochemical staining were performed to detect protein levels.

### Cell transfection

RNA oligonucleotides were transfected by Lipofectamine RNAiMAX (13778150, Invitrogen, Carlsbad, CA, USA); 50 nM RNA duplex (miRNA mimics and siRNAs) and 100 nM miRNA inhibitors were used as final concentration unless otherwise indicated. Lipofectamine 3000 (L3000015, Invitrogen) was used for plasmid transfection alone or co-transfection with RNA oligonucleotides.

### Luciferase reporter assay

AML12 (1.5 × 10^4^) or HEK293T cells (3 × 10^4^) grown in a 48-well plate were cotransfected with 50 ng (AML12) or 10 ng (HEK293T) *Firefly* luciferase reporter that carried indicated 3′UTR, 25 ng (AML12) or 5 ng (HEK293T) *Renilla* luciferase reporter pRL-TK (Promega, Madison, WT), together with 2.5 nM RNA duplex for 48 h before analysis. The luciferase assay was performed as reported.^48^ The *Firefly* luciferase activity of each sample was normalized to the *Renilla* luciferase activity.

### Lentivirus production and infection

The lentivirus expression vector pCDH-HSD3B7 that contained the target sequence of human HSD3B7 or its control vector pCDH-ctrl was co-transfected with Lenti-X HTX packing plasmids (Clontech, Palo Alto, CA, USA) into HEK293T cells by Lipofectamine 3000. After 72 h, the lentivirus supernatants were harvested and stored in aliquots at −80°C until use.

For infection, HepG2 cells were grown to 40% confluence and then infected with lentivirus supernatants supplemented with 10 μg/mL polybrene (TR-1003, Sigma-Aldrich) for 24 h and were selected with 2 μg/mL puromycin (HY-B1743A, MedChemExpress, Shanghai, China).

### Cell counting assay

For overexpressing of HSD3B7, 1.5 × 10^4^ (HepG2-Vector and HepG2-HSD3B7) or 3 × 10^4^ (Huh-7) viable cells were seeded into a 24-well plate and cultured for 4 days before cell counting. For TCA treatment assay, 2 × 10^4^ viable cells were seeding for 24 h, and then 25 μM taurocholic acid (TCA) (T4009, Sigma-Aldrich) were supplemented for another 4 days. For the administration of conditioned medium (CM, supernatants from AML12), 2 × 10^4^ viable tumor cells were seeded for 24 h before 20% of indicated CM were added and treated for another 4 days.

### Ethynyl deoxyuridine assay

Twenty-four hours after seeding into a 48-well plate with 1.5 × 10^4^ cells, Huh-7 cells were cultured in serum-free medium for 48 h, and then grew in 50% CM (from HepG2 cells) for 16 h, followed by labeling of 50 μM ethynyl deoxyuridine (EdU) for 2 h. The proportion of DNA-replicating cells was examined by EdU detection kit (C10310-1, RIOBIO) according to the instructions. The EdU incorporation rate was calculated as the ratio of the number of EdU positive cells to the number of Hoechst 33342-staining cells. At least 1,000 cells were counted for each group.

### Patients and human specimens

Healthy serum samples without HCC were obtained from patients who underwent physical examination without abnormal tumor biochemistry and ultrasound imaging in the First Affiliated Hospital of Sun Yat-sen University. Serum samples, HCC tumor tissues, and paracancerous tissues of HCC patients were collected from patients who underwent radical tumor resection at Sun Yat-sen University Cancer Center. Normal liver tissues were obtained from patients who underwent resection of hepatic hemangiomas at Sun Yat-sen University Cancer Center. No local or systemic treatment had been conducted before surgery. Informed consent was obtained from each patient, and the protocol was approved by the Sun Yat-sen University Institutional Research Ethics Committee. All tissues were examined histologically and immediately snap-frozen in liquid nitrogen until use.

### Bioinformatics and statistics

GSEA was performed to analyze the enrichment of the predefined sets of genes (molecular signature database, MSigDB) in the livers from *Mir122* KO and their wild-type littermates (accession no: GSE97060), as well as tumor tissues and paracancerous tissues from human HCC (TCGA dataset). The *q* and *p* values were examined by Kolmogorov-Smirnov statistic with GSEA v4.2.

To identify miR-122 targeted genes, the 3′UTR of indicated genes involved in BA synthesis (KEGG map 00120) were predicted by using either TargetScan (https://www.targetscan.org/vert_80/) for screening canonical sites or by alignment with RUGACUCC for searching non-canonical sites.^24^ And the expression of candidates in the liver was confirmed using The Human Protein Atlas (HPA; https://www.proteinatlas.org/). The Argonaute crosslinking immunoprecipitation (AGO-CLIP) sequencing data were obtained from GEO datasets (accession no: GSE97058).

The data from at least three independent experiments are presented as the mean ± SD unless other indicated. A *p* value of less than 0.05 was considered statistically significant. All statistical tests were conducted using GraphPad Prism 8.0 (GraphPad Software, Inc., San Diego, CA, USA). Unless otherwise noted, the differences between groups were analyzed using two-tailed unpaired or paired Student’s t test when only two groups or assessed by one-way ANOVA when more than two groups were compared. Correlation was explored by Pearson correlation coefficient. The minimum *p* value approach was used to separate the miR-122 high group from the miR-122 low group in the paracancerous tissues of 50 HCC patients (TCGA dataset), and Log rank (Mantel-Cox) test was used for Kaplan-Meier survival curve analysis to identify the prognostic factors.

## Data availability

RNA-seq and AGO-CLIP-seq data were deposited into the Gene Expression Omnibus database under accession number GSE97060 and GSE97058, respectively, which are available at the following URLs: https://www.ncbi.nlm.nih.gov/geo/query/acc.cgi?acc=GSE97060 and https://www.ncbi.nlm.nih.gov/gds/?term=GSE97058.

## Acknowledgments

We thank Prof. Shi-Mei Zhuang in Sun Yat-sen University for fruitful discussion and Chun-Xian Zeng, Kai You, and Jin-Feng Li in Sun Yat-sen University for technical assistance.

This work was funded by 10.13039/501100012166National Key R&D Program of China (2022YFA1303302), 10.13039/501100001809National Natural Science Foundation of China (32170782, 32100616), Science and Technology Projects in Guangzhou (202201010813), 10.13039/501100021171Guangdong Basic and Applied Basic Research Foundation (2023A1515012322, 2024A1515011089), and 10.13039/501100002858China Postdoctoral Science Foundation (2021M693670).

## Author contributions

J.H.H. designed and performed experiments, discussed and interpreted the data, and wrote the manuscript. Y.H.L., J.Z.H., R.N.L., R.W., Z.Q.C., S.Y.L., Y.L.C., and J.Y.H. performed experiments and interpreted the data. Y.Z. supervised and designed the study, discussed and interpreted the data, and wrote the manuscript. All authors read and approved the final manuscript.

## Declaration of interests

The authors declare no competing interests.

## Supplemental information

Document S1. Figures S1–S8, Tables S1–S2 and supplemental materials and methods

Document S2. Article plus supplemental information
