# Supplementary material for: Bile acid accumulation induced by miR-122 deficiency in liver parenchyma promotes cancer cell growth in hepatocellular carcinoma
Source: Mol Ther Nucleic Acids. 2025 May 14;36(2):102560. doi: 10.1016/j.omtn.2025.102560 (PMC12166698; doi:10.1016/j.omtn.2025.102560)
Supplement: Document S1. Figures S1–S8, Tables S1–S2 and supplemental materials and methods [file mmc1.pdf]

OMTN, Volume *36*

Supplemental information

Bile acid accumulation induced by miR-122 deficiency in liver parenchyma promotes cancer cell growth in hepatocellular carcinoma

Jia-Hui Huang, Yi-Hang Li, Juan-Zhen Hong, Ruo-Nan Li, Ruizhi Wang, Zi-Qi Chen, Song-Yang Li, Ying-Lei Chi, Jin-Yu Huang, and Ying Zhu

Supplemental Materials and Methods

Primary cells

Mouse primary hepatocytes were isolated from male LKO and CTRL mice by collagenase perfusion and density gradient centrifugation as previous reported[1]. Briefly, adult male mice with 8 weeks of age were anesthetized and the livers were perfused sequentially with ethylene diamine tetraacetic acid (EDTA) containing D-Hank's buffer at the speed of 3.5 mL/minute for 5-7 minutes, then followed by 100 U/mL collagenase IV-containing Hank's buffer for 15 min. The perfused livers were transferred to ice cold D-Hank's buffer, minced, filtered through a 100 μm cell strainer and centrifuged at 50 g for 1 minute to collect the hepatocytes pellets. Then the pellets were re-suspended in D-Hank's buffer containing 50% Percoll (17089109, GE Healthcare, Chicago, IL, USA), and centrifuged at 50 g for 15 minutes to collect the viable hepatocytes in pellets.

Plasmid construction

To identify miR-122 binding sites in the 3'UTR of Hsd3b7 or Akr1d1, the luciferase reporter plasmids named pGL3cm-human-HSD3B7-3'UTR-WT, pGL3cm-mouse-Hsd3b7-3'UTR-WT, and pGL3cm-mouse-Akr1d1-3'UTR-WT, were created by cloning 3'UTR fragments of human HSD3B7 (454-1961 nt; NM_025193.4), mouse Hsd3b7 (1064-1674 nt; NM_133943.3), or mouse Akr1d1 (1051-1231 nt; NM_145364.2), which contains putative binding sites of miR-122, into the *Eco*RI and *Xba*I sites downstream of the stop codon of *Firefly* luciferase in pGL3cm[2], which was produced based on pGL3-control (Promega). The

pGL3cm-human-HSD3B7-3'UTR-MUT and pGL3cm-mouse-Hsd3b7-3'UTR-MUT plasmids, which carried the mutated sequences in the complementary sites for the seed region of miR-122 were generated by fusion PCR based on the corresponding pGL3cm-3'UTR-WT vectors.

To generate pT3-EF1aH-mmu-miR-122-precursor and pT3-EF1aH-HSD3B7, 523 bp DNA fragment encompassing the mmu-miR-122 precursor sequence and its 5' and 3' flanking regions (230 bp and 232 bp, respectively), or the coding sequence of mouse Hsd3b7 (NM_001040684.2), was cloned into the *Eco*RV and *Not*I sites of pT3-EF1aH.

To generate the lentivirus vector pCDH-HSD3B7, the coding sequence of human HSD3B7 (NM_025193.4) was inserted into the *Eco*RI and *Xba*I sites of pCDH-CMV-MCS-EF1-copGFP (pCDH, System Biosciences), which includes a copGFP expression cassette.

All constructs were verified by direct DNA sequencing. All oligonucleotide sequences are listed in Table S2.

Analysis of gene expression

For Real-time quantitative polymerase chain reaction (qPCR) and Northern blotting analyses, total RNA was extracted by TRIzol reagent (15596018, Invitrogen). qPCR and Northern blotting were performed as described[3]. Reverse-transcribed using M-MLV reverse transcriptase (M1701, Promega). qPCR was performed on LightCycler 480 (Roche Diagnostic, Germany) using 2 x SYBR Green qPCR Master Mix (11201ES08, YEASEN,

Shanghai, China). The primers used for determination of miR-122 expression were designed by RIBOBIO, and other primers were purchased from TSINGKE (Guangzhou, China). All reactions were performed in duplicates. The cycle threshold (Ct) values differed by less than 0.5 between duplicates. All target genes were normalized to that of endogenous reference gene, which yielded a $2^{-\Delta\Delta Ct}$ value. Sequences for primers and probes were list in Table S2.

For immunoblotting analysis, tissues and total cell lysates were separated in SDS-polyacrylamide gels, electrophoretically transferred to polyvinylidene difluoride membranes (162-0177, Bio-Rad, Hercules, CA, USA), incubated sequentially with primary and secondary antibodies. The signal was developed with commercial ECL kit (1705061, Bio-Rad). The intensity of the specific band for target protein was detected using Image J software (Media Cybernetics, Bethesda, MD, USA). The antibodies for immunoblotting analysis were shown as following: rabbit polyclonal antibody against HSD3B7 (DF3653, affinity, Jiangsu, China), mouse monoclonal antibody against CYP7A1 (MABD42, Sigma-Aldrich), rabbit monoclonal antibody against CYP27A1 (ab126785, abcam, Cambridge, UK), rabbit polyclonal antibody against GAPDH (BA2913, BOSTER, California, USA); rabbit monoclonal antibody against β-actin (4970, Cell Signaling Technology, Beverly, MA, USA).

For immunohistochemical staining, formalin-fixed, paraffin-embedded tissues were cut into 3.5 μm sections, placed on polylysine-coated slides, dewaxed, quenched for endogenous peroxidase activity in 0.3% hydrogen peroxide, and processed for antigen

retrieval by high-pressure heating in sodium citrate buffer (pH = 6.0) for 10 minutes, followed by incubation with primary antibodies at 4 °C overnight. Rabbit monoclonal antibody against Ki-67 (12202, Cell Signaling Technology), goat polyclonal antibody against MMR / CD206 (AF2535, R&D SYSTEMS, Minnesota, USA), mouse monoclonal antibody against CYP7A1 and rabbit monoclonal antibody against α-SMA (ab124964, abcam) were used at the dilution of 1:1000, 1:200, 1:1000, and 1:1000 respectively. Immunostaining was performed using ChemMate DAKO EnVision Detection Kit, Peroxidase / DAB, Rabbit / Mouse (K5007, Agilent Technologies, Inc, CA, USA), which resulted in a brown-colored precipitate at the antigen site. Subsequently, sections were counterstained with hematoxylin (DH0005, leagene, Beijing, China) and mounted in non-aqueous mounting medium. All runs included a no primary antibody control. The stained sections were then scanned using a digital scanner (Aperio VERSA 200, Leica, Germany). The Ki-67, CD206, CYP7A1, and α-SMA staining area relative to the total tissue was evaluated using the Aperio software (Leica).

H&E and Sirius Red staining

Tissues were fixed in 4% paraformaldehyde and embedded in paraffin. H&E staining and Sirius Red staining of liver sections were performed as reported[1]. The collagen staining area relative to the total section area was evaluated using the Aperio software.

Serological analysis

Mouse serum was collected, centrifuged and measured enzymatically using alkaline phosphatase assay kit (A059-2-2, Nanjing Jiancheng Bioengineering Institute) and alanine aminotransferase assay kit (C009-2-1, Nanjing Jiancheng Bioengineering Institute) for the detection of AKP and ALT.

Table S1. List of 22 Bile Acids

No.	Bile Acids	Abbreviation	Formula
1	Cholic acid	CA	$C_{24}H_{40}O_5$
2	Glycocholic acid	G-CA	$C_{26}H_{43}NO_6$
3	Taurocholic acid	T-CA	$C_{26}H_{45}NO_7S$
4	Chenodeoxycholic acid	CDCA	$C_{24}H_{40}O_4$
5	Taurochenodeoxycholic acid	T-CDCA	$C_{26}H_{45}NO_6S$
6	α-Muricholic acid	αMCA	$C_{24}H_{40}O_5$
7	β-Muricholic acid	βMCA	$C_{24}H_{40}O_5$
8	Tauro α-Muricholic acid	T-αMCA	$C_{26}H_{45}NO_7S$
9	Tauro β-Muricholic acid	T-βMCA	$C_{26}H_{45}NO_7S$
10	Ursodeoxycholic acid	UDCA	$C_{24}H_{40}O_4$
11	Tauroursodeoxycholic acid	T-UDCA	$C_{26}H_{45}NO_6S$
12	Deoxycholic acid	DCA	$C_{24}H_{40}O_4$
13	Taurodeoxycholic acid	T-DCA	$C_{26}H_{45}NO_6S$
14	Lithocholic acid	LCA	$C_{24}H_{40}O_3$
15	Taurolithocholic acid	T-LCA	$C_{26}H_{45}NO_5S$
16	Hyodeoxycholic acid	HDCA	$C_{24}H_{40}O_4$
17	Taurohyodeoxycholic acid	T-HDCA	$C_{26}H_{45}NO_6S$
18	7-Ketodeoxycholic acid	7-DHCA	$C_{24}H_{38}O_5$
19	12-Dehydrocholic acid	12-DHCA	$C_{24}H_{38}O_5$
20	6,7-Diketolithocholic acid	6,7-diketoLCA	$C_{24}H_{36}O_5$
21	Allolithocholic acid	alloLCA	$C_{24}H_{40}O_3$
22	Allocholic acid	ACA	$C_{24}H_{40}O_5$

Table S2. Sequence of Oligonucleotides

Name	Sense Strand / Sense Primer (5'-3')	Antisense Primer (5'-3')
**miRNA mimics**		
miR-122	UGGAGUGUGACAAUGGUGUUUG	AACACCAUUGUCACACUCAUUU
NC	UCACAACCUCCUAGAAAGAGUAGA	UACUCUUUCUAGGAGGUUGUUAUU
**miRNA inhibitors**		
anti-miR-122	CAAACACCAUUGUCACACUCCA	
anti-NC	GUGGAUAUUGUUGCCAUCA	
**siRNA duplexes**		
siHSD3B7-1(human)	CAGAAUGGCUGUCCUUGUCGUdTdT	ACGACAAGGACAGCCAUUCUGdGdC
siHSD3B7-2(human)	GUGUUCCUGGCUGCCCUCAAUdTdT	AUUGAGGGCAGCCAGGAACACdCdA
**Primers for RT-qPCR**		
Cyp7a1(mouse)	TCACAAACTCCCTGTCATACCA	ATCTCCCTGGAGGGTTTTGT
Cyp8b1 (mouse)	TGAATTCTTGAAGGGGATGC	GGTACCCAAACACCTTGAGC
Cyp27a1 (mouse)	GGACCGGAACGCTACAATTT	ACTTGCCCTCCTGTCTCATC
Cyp7b1 (mouse)	GCTTCCTTATCTTGGCATGG	ATCGGCTGCTGAACTTCTGA
Hsd3b7 (mouse)	TGATTGATGCTTGTGTGCAG	CTGCTCAGCAAGGGCTTTAC
Akr1d1 (mouse)	AAGACAGCTATTGATGAGGGGT	CCTCTTTACCTTCCCTTCTGCTA
Abcb11 (mouse)	CTGCCAAGGATGCTAATGCA	CGATGGCTACCCTTTGCTTCT
Abcc2 (mouse)	GGATGGTGACTGTGGGCTGAT	GGCTGTTCTCCCTTCTCATGG
Abcc3 (mouse)	TCCCACTTTTCGGAGACAGTAAC	ACTGAGGACCTTGAAGTCTTGGA
Slc51a (mouse)	TGTTCCAGGTGCTTGTCATCC	CCACTGTTAGCCAAGATGGAGAA
Slc51b (mouse)	GATGCGGCTCCTTGGAATTA	GGAGGAACATGCTTGTCATGAC
Slc10a1 (mouse)	ATGACCACCTGCTCCAGCTT	GCCTTTGTAGGGCACCTTGT
Slco1a1 (mouse)	CAGTCTTACGAGTGTGCTCCAGAT	ATGAGGAATACTGCCTCTGAAGT
Actb (mouse)	CCCTGAAGTACCCCATTGAA	CTTTTCACGGTTGGCCTTAG
Gapdh (mouse)	AACTTTGGCATTGTGGAAGG	CACATTGGGGGTAGGAACAC

Table S2. Sequence of Oligonucleotides (Continued)

**Name**	**Sense Strand / Sense Primer (5'-3')**	**Antisense Primer (5'-3')**
Il-6 (mouse)	TTCAACCAAGAGGTAAAAGATTTACA	CACTCCTTCTGTGACTCCAGCT
Ccl2 (mouse)	TCTGTGCTGACCCCAAGAAGG	TGGTTGTGGAAAAGGTAGTGGAT
HSD3B7 (human)	CTGGGCTGGTAGACGTGTTT	TGTCTTCGTTGCCCCTGTAG
ACTB (human)	ACTGGAACGGTGAAGGTGAC	AGAGAAGTGGGGTGGCTTTT
**Primers for cloning (restriction enzyme sites were underlined)**		
human HSD3B7-3'UTR	CCGGAATTCTCAAGCAATCCTCCTGCC	TGCTCTAGA GGACTGAGGTGGCTGGAG
mouse Hsd3b7-3'UTR	CCGGAATTCGTACCAACAAGGCACAGCGG	TGCTCTAGAGGAGAGGCAAGACATGGGAA
mouse Akr1d1-3'UTR	CCGGAATTCACATGGAAATTTCTTCAACAGAG	TGCTCTAGATCTTCTTTTCTCTTCATCAGAGC
mmu-miR-122 precursor	GGCACGCGATATCTGTAAGAAGTGTCTGCCTC	ATAAGAATGCGGCCGCGAGGGTCCCATAGGA GAGG
mouse Hsd3b7	ATAAGAATGATATCGCCACCATGCTGCTGGAACGGGAG	ATATTAGCGGCCGCTCACCGAGCTGAACCCTC CATC
human HSD3B7	CCGGAATTCATGGCCGACTCTGCACAG	TGCTCTAGATCACTGGGCTGAACCC GTAG
**Probes for Northern blotting**		
miR-122	CAAACACCATTGTCACACTCCA	
U6	AACGCTTCACGAATTTGCGT	

Supplemental Figures and Legends

Figure S1

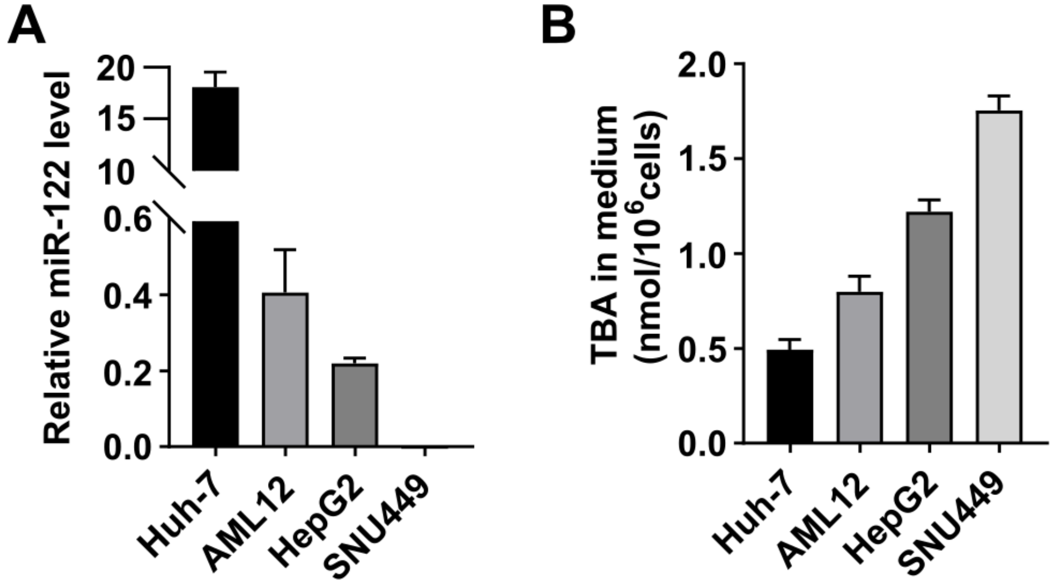

Figure S1. The level of BAs was negatively related to the expression of miR-122 in hepatoma, hepatoblastoma and normal hepatocytes cell lines. Related to Figure 1.

(A) The endogenous miR-122 level was detected in human hepatoma cell lines (Huh-7 and SNU449), hepatoblastoma cell line (HepG2) and mouse hepatocyte cell line AML12. (B) The concentrations of secreted TBA were analyzed in different cell lines. Data from at least three independent experiments are shown as mean ± SD.

Figure S2

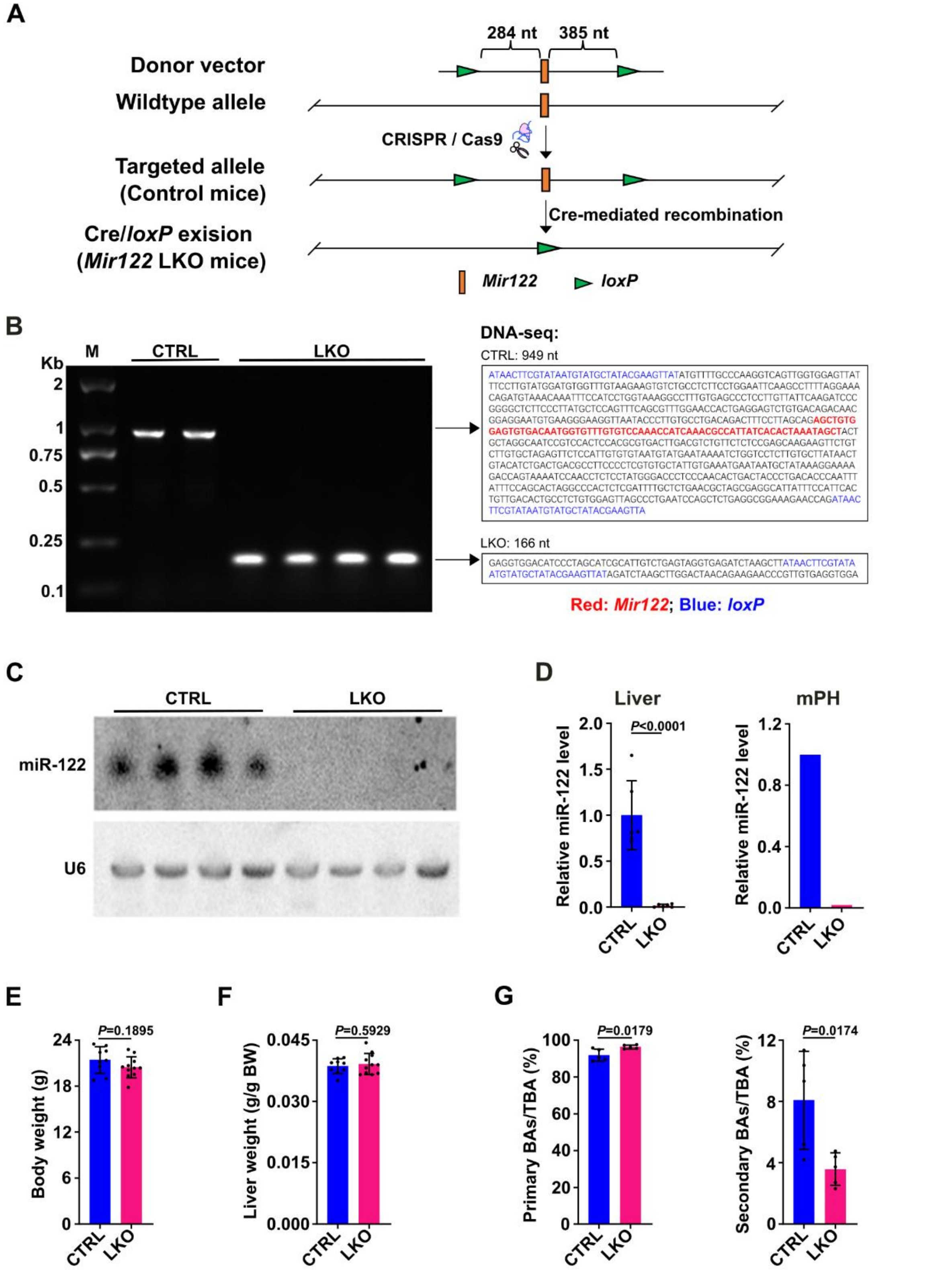
A
284 nt
385 nt
Donor vector
Wildtype allele
CRISPR / Cas9
Targeted allele
(Control mice)
Cre-mediated recombination
Cre/loxP exision
(Mir122 LKO mice)
Mir122
loxP
B
M
CTRL
LKO
Kb
2
1
0.75
0.5
0.25
0.1
DNA-seq:
CTRL: 949 nt
ATAACTTCGTATAATGTATGCTATACGAAGTTATATGTTTTGCCCAAGGTCAGTTGGTGGAGTTAT
TTCCTTGTATGGATGTGGTTTGTAAGAAGTGTCTGCCTCTTCCTGGAATTCAAGCCTTTTAGGAAA
CAGATGTAAACAAATTTCCATCCTGGTAAAGGCCTTTGTGAGCCCTCCTTGTTATTCAAGATCCC
GGGGCTCTTCCCTTATGCTCCAGTTTCAGCGTTTGGAACCACTGAGGAGTCTGTGACAGACAAC
GGAGGAATGTGAAGGGAAGGTTAATACCCTTGTGCCTGACAGACTTTCCTTAGCAGAGCTGTG
GAGTGTGACAATGGTGTTTGTGTCCAAACCATCAAACGCCATTATCACACTAAATAGCTACT
GCTAGGCAATCCGTCCACTCCACGCGTGACTTGACGTCTGTTCTCTCCGAGCAAGAAGTTCTGT
CTTGTGCTAGAGTTCTCCATTGTGTGTAATGTATGAATAAAATCTGGTCCTCTTGTGCTTATAACT
GTACATCTGACTGACGCCTTCCCCTCGTGTGCTATTGTGAAATGAATAATGCTATAAAGGAAAA
GACCAGTAAAATCCAACCTCTCCTATGGGACCCTCCCAACACTGACTACCCTGACACCCAATTT
ATTTCCAGCACTAGGCCCACTCTCGATTTTGCTCTGAACGCTAGCGAGGCATTATTTCCATTCAC
TGTTGACACTGCCTCTGTGGAGTTAGCCCTGAATCCAGCTCTGAGGCGGAAAGAACCAGATAAC
TTCGTATAATGTATGCTATACGAAGTTA
LKO: 166 nt
GAGGTGGACATCCCTAGCATCGCATTGTCTGAGTAGGTGAGATCTAAGCTTATAACTTCGTATA
ATGTATGCTATACGAAGTTATAGATCTAAGCTTGGACTAACAGAAGAACCCGTTGTGAGGTGGA
Red: Mir122; Blue: loxP
C
CTRL
LKO
miR-122
U6
D
Liver
mPH
Relative miR-122 level
P<0.0001
2.0
1.5
1.0
0.5
0.0
1.2
0.8
0.4
0.0
CTRL
LKO
E
Body weight (g)
P=0.1895
24
18
12
6
0
CTRL
LKO
F
Liver weight (g/g BW)
P=0.5929
0.045
0.030
0.015
0.000
CTRL
LKO
G
Primary BAs/TBA (%)
P=0.0179
100
80
60
40
20
0
Secondary BAs/TBA (%)
P=0.0174
12
8
4
0
CTRL
LKO

Figure S2. Generation of liver specific *Mir122* knockout mice. Related to Figure 2.

(A) The strategy for generating conditional *Mir122* knockout in hepatocytes by CRISPR / Cas9. Cas9 mRNA, sgRNA and donor vector were co-injected into zygotes. sgRNA directed Cas9 endonuclease cleavage upstream and downstream of *Mir122* and created a double-strand break. Such breaks were repaired, and resulted in *loxP* sites inserted in the upstream and downstream of *Mir122* respectively by homologous recombination. *Mir122* LKO allele were obtained after Cre-mediated recombination. (B) DNA sequencing analysis of liver DNA from indicated genotypes. We first extracted liver DNA from LKO and CTRL mice and amplified the DNA fragment between the two *loxP* sites by PCR, followed by gel electrophoresis analysis and DNA sequencing. CTRL, 949 nt; LKO, 166 nt. (C) Northern blotting analysis of hepatic miR-122 from CTRL and LKO mice. (D) miR-122 expression in liver tissues and mouse primary hepatocytes (mPH) from CTRL and LKO mice was determined by qPCR. (E, F) The body weight (E) and the ratio of liver weight to body weight (BW) (F) were similar between LKO mice and their control littermates at the age of 8 weeks (n=9-11 mice per group). (G) The proportion of hepatic primary BAs and secondary BAs was detected in LKO mice (n=5 mice per group). Hepatic individual BA levels of CTRL and LKO mice were determined by UHPLC-MS / MS, as presented in Figure 2(C)-(F). For (C) and (D), U6 was used as internal control. The data are presented as mean ± SD; *p* values were assessed by unpaired Student's *t*-test.

Figure S3

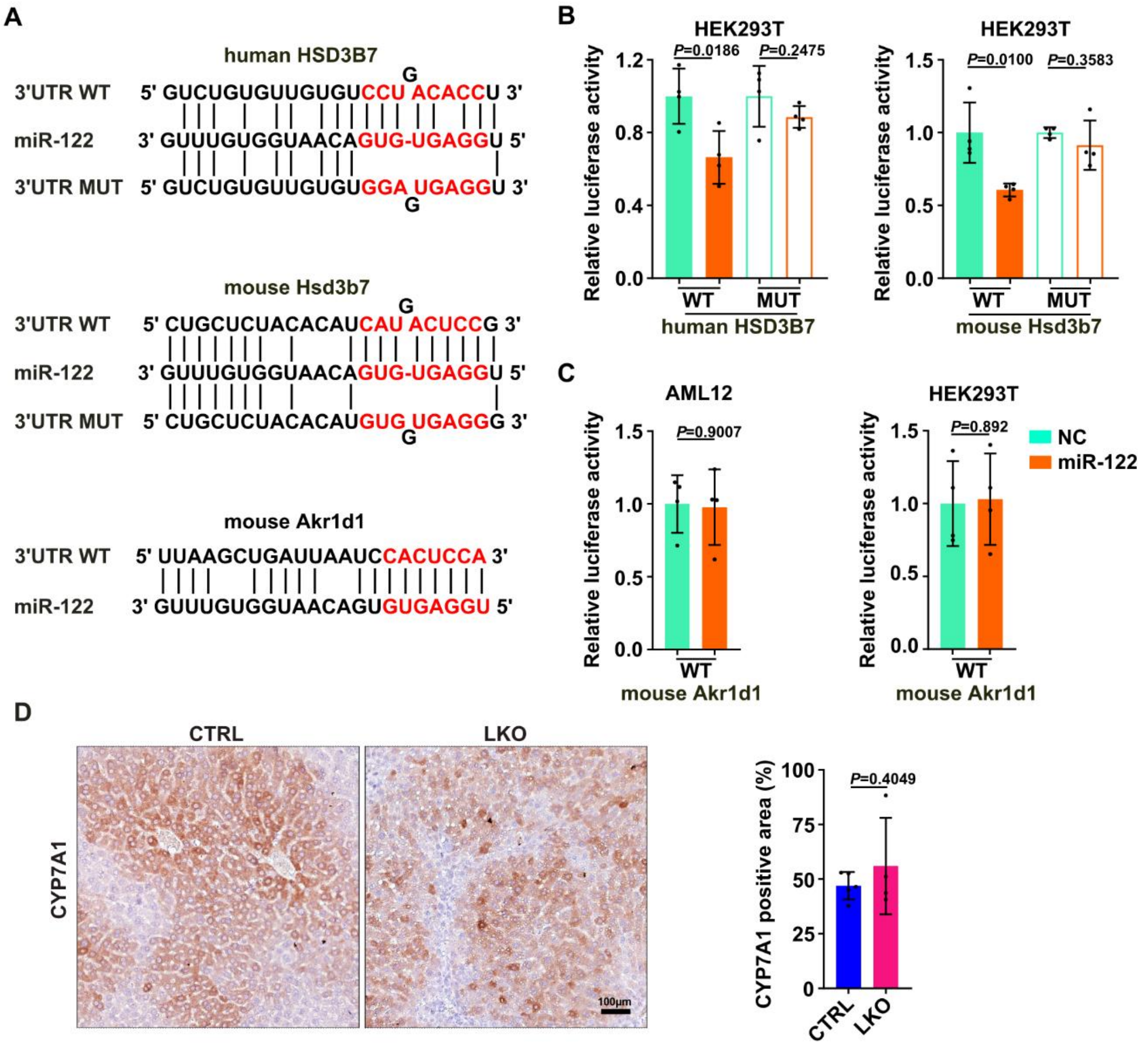

Figure S3. Screening for the targets of miR-122. Related to Figure 3.

(A) Wild-type miR-122 sequence, wild-type and mutant 3'UTR segments of human HSD3B7, mouse Hsd3b7 or Akr1d1 are shown. Mutations were generated in the complementary site (in red) that binds to the seed region (in red) of miR-122. (B, C) The luciferase activities of the reporters containing the wildtype or mutant HSD3B7 3'UTR from both human and mouse, or containing the mouse wildtype Akr1d1 3'UTR were examined

in HEK293T and AML12. (D) BA synthesis enzyme CYP7A1 was unchanged in LKO livers, which was determined by IHC (n=4-5 mice per group). Scale bar, 100μm. The data are presented as mean ± SD; *p* values were examined by unpaired Student's *t*-test (B, C and D, left panel).

Figure S4

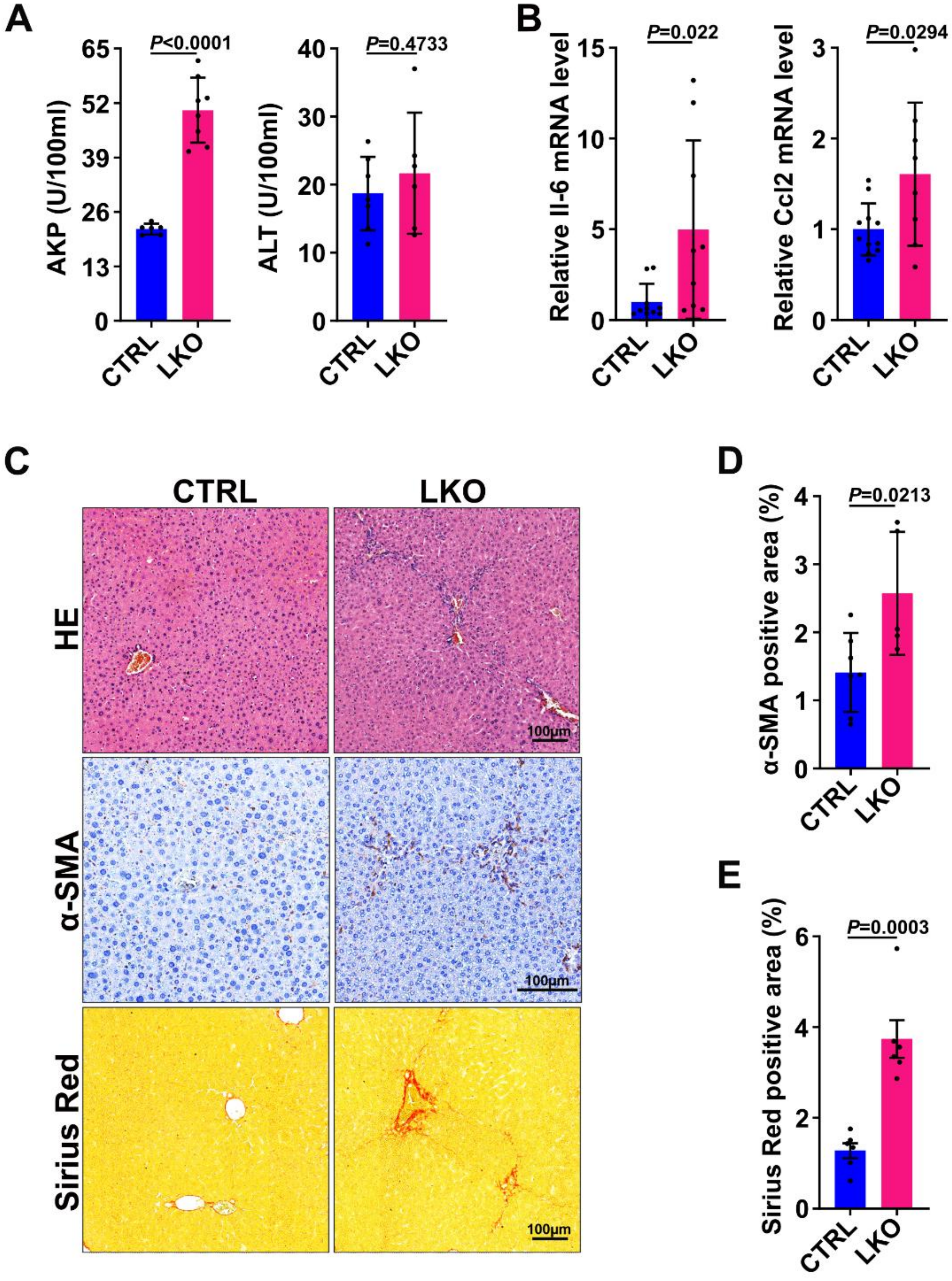
A
AKP (U/100ml)
P<0.0001
0
13
26
39
52
65
CTRL
LKO
ALT (U/100ml)
P=0.4733
0
10
20
30
40
B
Relative Il-6 mRNA level
P=0.022
0
5
10
15
Relative Ccl2 mRNA level
P=0.0294
0
1
2
3
C
CTRL
LKO
HE
α-SMA
Sirius Red
100μm
D
α-SMA positive area (%)
P=0.0213
0
1
2
3
4
E
Sirius Red positive area (%)
P=0.0003
0
2
4
6

Figure S4. LKO mice exhibit phenotypes of liver injury, inflammation and fibrosis. Related to Figure 5.

(A) Higher level of serum AKP was observed in LKO mice (n=6-8 mice per group). Serum alkaline phosphatase (AKP) and alanine aminotransferase (ALT) were measured enzymatically in 12-week-old male mice. (B) The expression of Il-6 and Ccl2 was detected by qPCR in LKO livers when mice were 33 weeks old (n=8-11 mice per group). (C-E) LKO male mice developed hepatic fibrosis at the age of 33 weeks, which were determined by staining of α-SMA (C, D) and Sirius Red (C, E) (n=5-7 mice per group). Scale bars, 100 μm. The data are presented as mean ± SD; *p* values were assessed by unpaired Student's *t* test.

Figure S5

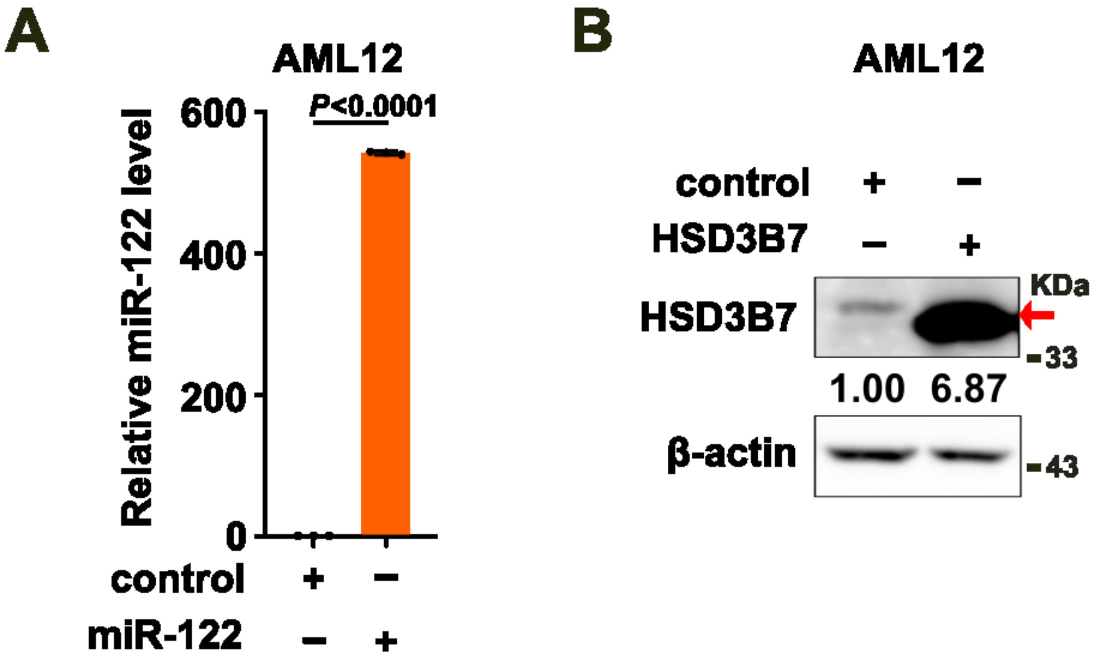

Figure S5. Validation the overexpression effect of pT3-EF1aH-mmu-miR-122-precursor and pT3-EF1aH-HSD3B7 in AML12 cell line. Related to Figure 6.

(A) Validation the overexpression effect of pT3-EF1aH-mmu-miR-122-precursor. (B) Validation the overexpression effect of pT3-EF1aH-HSD3B7. (A-B) AML12 cells were transfected with pT3-EF1aH-mmu-miR-122-precursor, or pT3-EF1aH-HSD3B7, or pT3-EF1aH (named control) for 48 hours prior to qPCR or western blotting. "+" or "-", presence (+) or absence (-) of the treatment. The target protein relative to β-actin were measured by Image J, which is indicated under each band. The data from at least three independent experiments are presented as mean ± SD; *p* value was determined by unpaired Student's *t*-test (A).

Figure S6

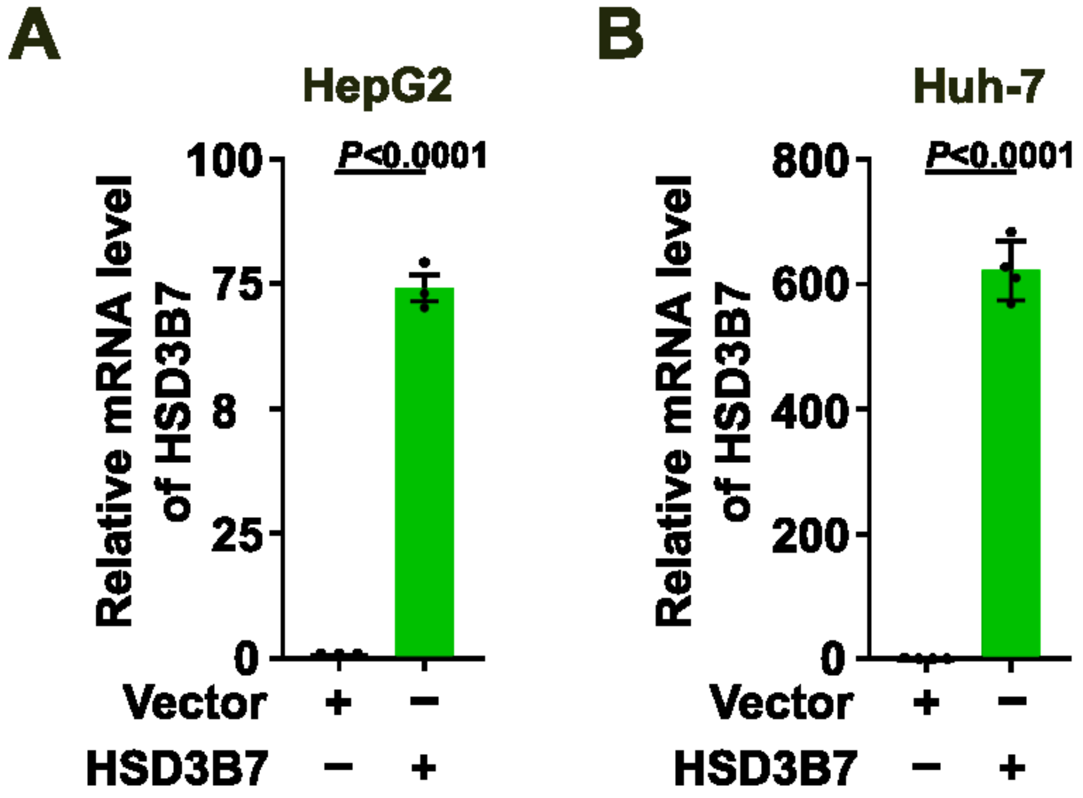

Figure S6. The effect of overexpression of HSD3B7. **Related to Figure 7.**

(A) The mRNA levels of HepG2-HSD3B7 and HepG2-Vector subline were measured by qPCR. (B) The mRNA level of HSD3B7 were measured after overexpressing of HSD3B7 in Huh-7. Huh-7 cells were transfected with pCDH-HSD3B7 or its control vector for 48 hours prior to qPCR. The data are presented as mean ± SD; *p* values were examined by unpaired Student's *t*-test.

Figure S7

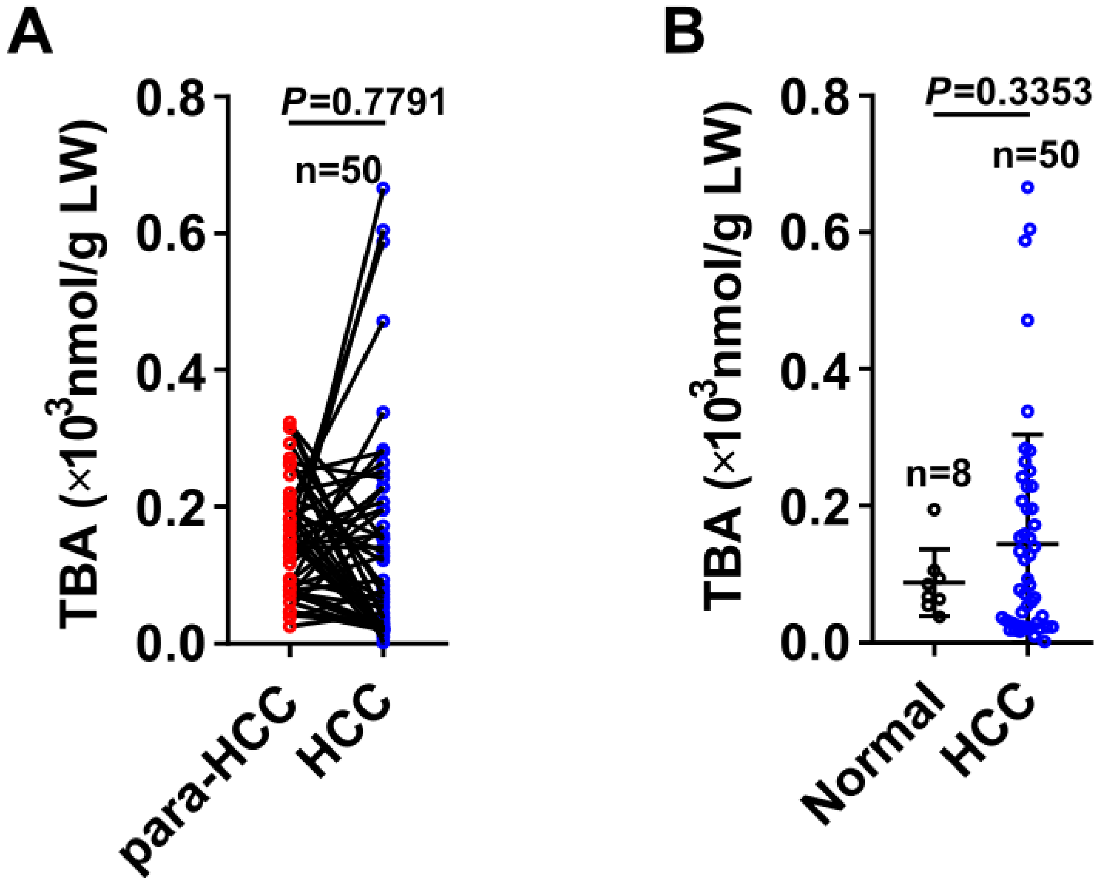

Figure S7. TBA level is not dramatic induction in HCC tumor tissues, compared with paired paracancerous tissues or normal liver tissues. Related to Figure 8.

TBA level was examined in 8 normal livers, 50 paired HCC tumor tissues and paracancerous tissues (para-HCC). *p* values were assessed by 2-tailed paired Student's *t* test (A) or unpaired Student's *t* test (B).

Figure S8

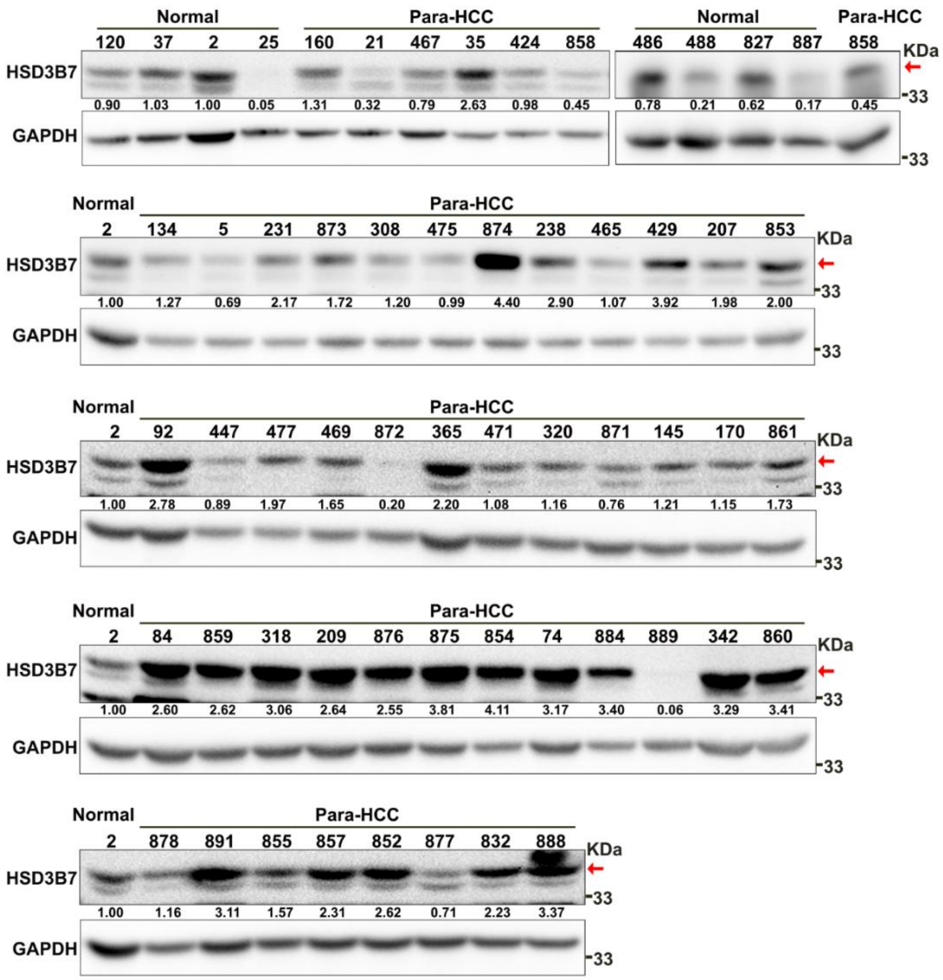

Figure S8. The upregulation of HSD3B7 protein level in the paracancerous tissues from HCC patients. Related to Figure 8.

The protein level of HSD3B7 was detected in normal liver tissues from patients undergoing resection of hepatic hemangioma (n=8) (Normal) and paracancerous tissues from HCC patients (para-HCC) (n=50). The protein level of HSD3B7 relative to GAPDH is indicated under each band. Red arrow indicates the band of HSD3B7 protein.

Supplemental References

1. You K, Li S Y, Gong J, Fang J H, Zhang C, Zhang M, Yuan Y F, Yang J N, Zhuang S M. MicroRNA-125b Promotes Hepatic Stellate Cell Activation and Liver Fibrosis by Activating RhoA Signaling. Mol Ther Nucleic Acids 2018;12: 57-66.

2. Su H, Yang J R, Xu T, Huang J, Xu L, Yuan Y F, Zhuang S M. MicroRNA-101, down-regulated in hepatocellular carcinoma, promotes apoptosis and suppresses tumorigenicity. Cancer Res 2009;69:1135-1142.

3. Zeng C X, Wang R, Li D, Lin X J, Wei Q K, Yuan Y, Wang Q, Chen W, Zhuang S M.. A novel GSK-3 beta-C/EBP alpha-miR-122-insulin-like growth factor 1 receptor regulatory circuitry in human hepatocellular carcinoma. Hepatology 2010;*52*:1702-1712.
